# Intraspecific trait variability and community assembly in hawkmoths (Lepidoptera: *Sphingidae*) across an elevational gradient in the eastern Himalayas, India

**DOI:** 10.1002/ece3.7054

**Published:** 2021-02-25

**Authors:** Mansi Mungee, Ramana Athreya

**Affiliations:** ^1^ Indian Institute of Science Education and Research Pune India; ^2^ Wildlife Institute of India Dehradun India

**Keywords:** community assembly, intraspecific variance, invertebrates, *Sphingidae*, *T‐statistics*

## Abstract

1. We investigated some aspects of hawkmoth community assembly at 13 elevations along a 200‐ to 2770‐m transect in the eastern Himalayas, a little studied biodiversity hot spot of global importance. We measured the morphological traits of body mass, wing loading, and wing aspect ratio of 3,301 free‐ranging individuals of 76 species without having to collect or even constrain them. We used these trait measurements and *T‐statistic* metrics to assess the strength of intracommunity (“internal") and extra‐community (“external”) filters which determine the composition of communities *vis‐a‐vis* the regional pool of species.
2. The trait distribution of constituent species turned out to be nonrandom subsets of the community‐trait distribution, providing strong evidence for internal filtering in all elevational communities. The external filter metric was more ambiguous. However, the elevational dependence of many metrics including that of the internal filter provided evidence for external (i.e., environmental) filtering. On average, a species occupied as much as 50%–75% of the total community‐trait space, yet the *T‐statistic* metric for internal filter was sufficiently sensitive to detect a strong nonrandom structure in the trait distribution.
3. We suggest that the change in *T‐statistic* metrics along the environmental gradient may provide more clues to the process of community assembly than previously envisaged. A large, smoothly varying and well‐sampled environmental span would make it easier to discern them. Developing *T‐statistics* for combined analysis of multiple traits will perhaps provide a more accurate picture of internal/filtering and niche complementarity. Moths are a hyperdiverse taxon and a very important component of many ecosystems. Our technique for accurately measuring body and wing dimensions of free‐ranging moths can generate trait database for a large number of individuals in a time‐ and resource‐efficient manner for a variety of community assembly studies using this important taxon.

## Graphical abstract

We investigated the role of traits (body mass, wing loading, and wing aspect ratio) in the community assembly of hawkmoths (Lepidoptera: Sphingidae) using 3,301 individuals from 76 species, along an elevational gradient spanning 2,600 m in the eastern Himalayas. We found strong evidence for the role of both internal and external filters in hawkmoth community assembly. Our technique for accurately measuring body and wing dimensions of free‐ranging moths can generate trait database for a large number of individuals, which can be used in a variety of community assembly studies of a hyperdiverse taxon which is also a very important component of many ecosystems.

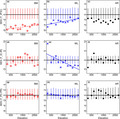

## 1 INTRODUCTION

Ecological processes which govern community assembly may be separated into two categories, those causing either a convergence or a divergence of functional traits of species co‐occurring in a community (e.g., Enquist et al., 2015; Grime, 2006; Weiher et al., 2011; Weiher & Keddy, 1995). The abiotic environment causes trait convergence by constraining the trait values of all species in a community to a range that facilitates their persistence in that habitat (e.g., Diaz et al., 1998; Weiher et al., 1998). On the other hand, traits of co‐occurring species are expected to diverge from each other to reduce ecological similarity and hence debilitating competition (MacArthur & Levins, 1967). Several metrics of functional (trait) diversity have been used to characterize the distribution of species mean traits in a community (Mouchet et al., 2010; Villéger et al., 2008), and detect signatures of community assembly processes (e.g., Ackerly, 2003; Baraloto et al., 2012; Bryant et al., 2008; Choler, 2005; Fonseca et al., 2000; Pigot et al., 2016; Swenson & Enquist, 2007). The importance of incorporating intraspecific trait variability (ITV) into such studies has been increasingly recognized over the last decade (e.g., Albert et al., 2011; Bolnick et al., 2011; Cianciaruso et al., 2009; Enquist et al., 2015; Hulshof et al., 2010; Jung et al., 2010; Paine et al., 2011).

However, biotic interactions like competitive exclusion (HilleRisLambers et al., 2012), equalizing fitness or facilitation (Butterfield & Callaway, 2013; Grime, 2006), and trait trade‐offs (e.g., Spasojevic & Suding, 2012) have signatures similar to abiotic filters, while microhabitat heterogeneity, an abiotic filter, can confound the signature of interspecific competition (Violle et al., 2012). Therefore, Violle et al. (2012) recast community assembly processes into two other categories: filters internal to the community (includes both biotic and abiotic: e.g., interspecific competition and microhabitat heterogeneity) and filters external to the community (both biotic and abiotic: e.g., climate and predators). Internal filters determine species coexistence within the community after the external filters have filtered a subset from the larger regional pool into the community.

Violle et al. (2012) proposed *T‐statistics*, a suite of three functional trait metrics, to identify the external and internal filters contributing to community assembly across a region. In their formulation, the “region” spans a range in environmental space, and each of the many “communities” which make up the region is collections of species (the taxon of interest) localized in small volumes within the regional environmental space. The *T‐statistic* metrics consist of variance ratios of functional traits across taxonomic (population, species, and community) and spatial (local and regional) scales to identify the operational filters. The metrics have been utilized in two ways. Their directional change along an environmental gradient is considered evidence of external filters (Allgeier et al., 2017; Hulshof et al., 2013; Le Bagousse‐Pinguet et al., 2014; Wu et al., 2019). Additionally, one can test for deviation of the measured values of the metrics from those expected from randomness. Significant deviation from randomness is considered evidence of the impact of an ecological process on the trait distribution (Danet et al., 2018; Gusmão et al., 2020; Khalil et al., 2019; Luo et al., 2016; Neyret et al., 2016; Outreman et al., 2018; Subedi et al., 2019; Xavier‐Jordani et al., 2019; Zorger et al., 2019).

Community assembly studies using *T‐statistics* require trait measurements of (many) individuals of a species and therefore have mostly targeted plants with only a small number of faunal studies: aphid parasitoids (Outreman et al., 2018), spiders (Gusmão et al., 2020), moths (Wu et al., 2019), and amphibians (Xavier‐Jordani et al., 2019). The relationship between traits and their functionality is more easily quantifiable in plants, and the traits are more easily measured for a large number of individuals (Lamanna et al., 2014; Lavorel et al., 2013), than is the case with faunal taxa (Brousseau et al., 2018). While museum samples do provide large repositories of specimens for trait measurements, they are seldom compiled through systematic sampling efforts; most collections are composites from multiple locations and periods.

The 13 studies of community assemblage using *T‐statistics* (all cited previously) differ in the taxa studied, nature of the gradient, and species richness. Yet, some trends are already visible: (a) In (almost) all cases, trait distributions within a community are nonrandom subsets, with individuals of a species clustered closer to each other than to other species, (b) communities may or may not be nonrandom subsets within the region; there is no consistent pattern either within a study or across different studies, and (c) the use of ITV accentuates the nonrandom nature of communities within the region in most studies. Other results, essentially correlations between the environment, *T‐statistic* metrics, and other community parameters (like species richness), varied across studies though not all studies investigated all possible correlations. Such correlations contain clues to the identity of the processes (e.g., niche v/s neutral) impacting community assembly (Violle et al., 2012).

Apart from those using *T‐statistics,* only a few studies have dealt with changes in trait distribution (of which variance is the simplest metric) with elevation (e.g., Baranovská & Knapp, 2018; Classen et al., 2017). In general, studies have reported increased variability in traits under “favorable conditions” at lower elevations where intra‐ and interspecific competition drives trait divergence (Ding et al., 2019; Mayfield & Levine, 2010), while habitat filtering due to extreme environmental conditions at higher elevations is associated with reduced trait variance (de Bello et al., 2009; Kraft & Ackerly, 2010).

We present here a study of the roles of internal and external filters in community assembly of hawkmoths in 13 elevational communities in the elevational range of 200–2770 m. We analyzed three key morphological traits (body mass, wing loading, and wing aspect ratio) in the *T‐statistics* framework and with measurements of 3,301 individual hawkmoths *(*Lepidoptera: *Sphingidae)* belonging to 76 species. We also investigated the change in community‐wide variance of these traits with elevation.

The eastern Himalayas are among the most biologically diverse regions in the world (Myers et al., 2000; Orme et al., 2005). Its large environmental gradient and biodiversity (of which moths are a prime example) make an excellent combination for investigating the link between environment and diversity. Very few ecological studies have been carried out there despite their global importance. New species, of even distinctive vertebrate taxa, continue to be described from the region (e.g., Athreya, 2006a; Captain et al., 2019; Mirza et al., 2020; Sinha et al., 2005; Sondhi & Ohler, 2011). The entire list of research publications on diversity patterns in the region is a short one: elevational gradient of bird diversity (Acharya et al., 2011; Price et al., 2014; Schumm et al., 2020; Surya & Keitt, 2019), tree diversity patterns and population structure (Bhuyan et al., 2003; Rana et al., 2019), and distribution and abundance of arthropods (Ghosh‐Harihar, 2013; Marathe et al., 2020; Supriya et al., 2020).

Moths are a hyperdiverse insect taxon (Quimbayo et al., 2010; Scoble & Hausmann, 2007), second only to Coleoptera. From our work, we expect over 2000 moth species across our elevational transect. On occasion, we have recorded over 2,500 individuals from more than 200 species on our sampling screens at a single location on a single night. This large species diversity and abundance provides opportunities to understand community assembly with sufficient statistical strength at multiple levels: within a genus, within a family, or across different families. Moth larvae are among the principal (most abundant) herbivores and prey base for insectivores in many ecosystems (Lill & Marquis, 2003; Supriya et al., 2020). The food–plant specificity of moth species makes for an intimate linkage between plant and moth communities. These factors make them an excellent system for long‐term monitoring to understand the cascading effect of climate change on primary producers and two trophic levels above them. We selected the hawkmoth family because as a group they are easier to separate from other moths, and identify to (morpho) species even from an image.

We selected the three traits of body mass, wing loading, and wing aspect ratio because (a) they impact multiple aspects of a moth's life history such as thermoregulation (Dillon et al., 2006; Dudley, 2002; Heinrich, 1996), dispersal (Athreya & Singh, 1990; Azevedo et al., 1998; Frazier et al., 2008; Gilchrist & Huey, 2004; Lentink et al., 2007; Rohner et al., 2018), reproduction (Moretti et al., 2017; Suding & Goldstein, 2008), and starvation resistance (Cushman et al., 1993; Lindsey, 1966) and therefore should be functional response traits, and (b) we were able to measure these traits from images of free‐flying moths without even momentarily constraining them, let alone collecting specimens (Mungee & Athreya, 2020).

Based on the previous discussion, we tested the following hypotheses in this study: Hawkmoth elevational communities are not random subsets but bear the imprint of internal and external filters (i.e., consequences of several ecological processes).

1. Internal filter: The distribution of trait values within a species in a community is not a random subset of the trait values of all the individuals (regardless of species) within that community.
2. External filter: The distribution of trait values of individuals of a community is not a random subset of the trait values of all the individuals within the region.

Additionally, we tested a related hypothesis associated with community‐trait variance:

1. Community‐trait variance should decrease toward higher elevations as the harsher conditions there should result in tighter constraints on trait dispersion.

We also tested for correlations between the metrics for internal and external filters on the one hand, and species richness and elevation on the other.

## 2 METHODS AND MATERIALS

### 2.1 Study area and field sampling

**FIGURE 1:**
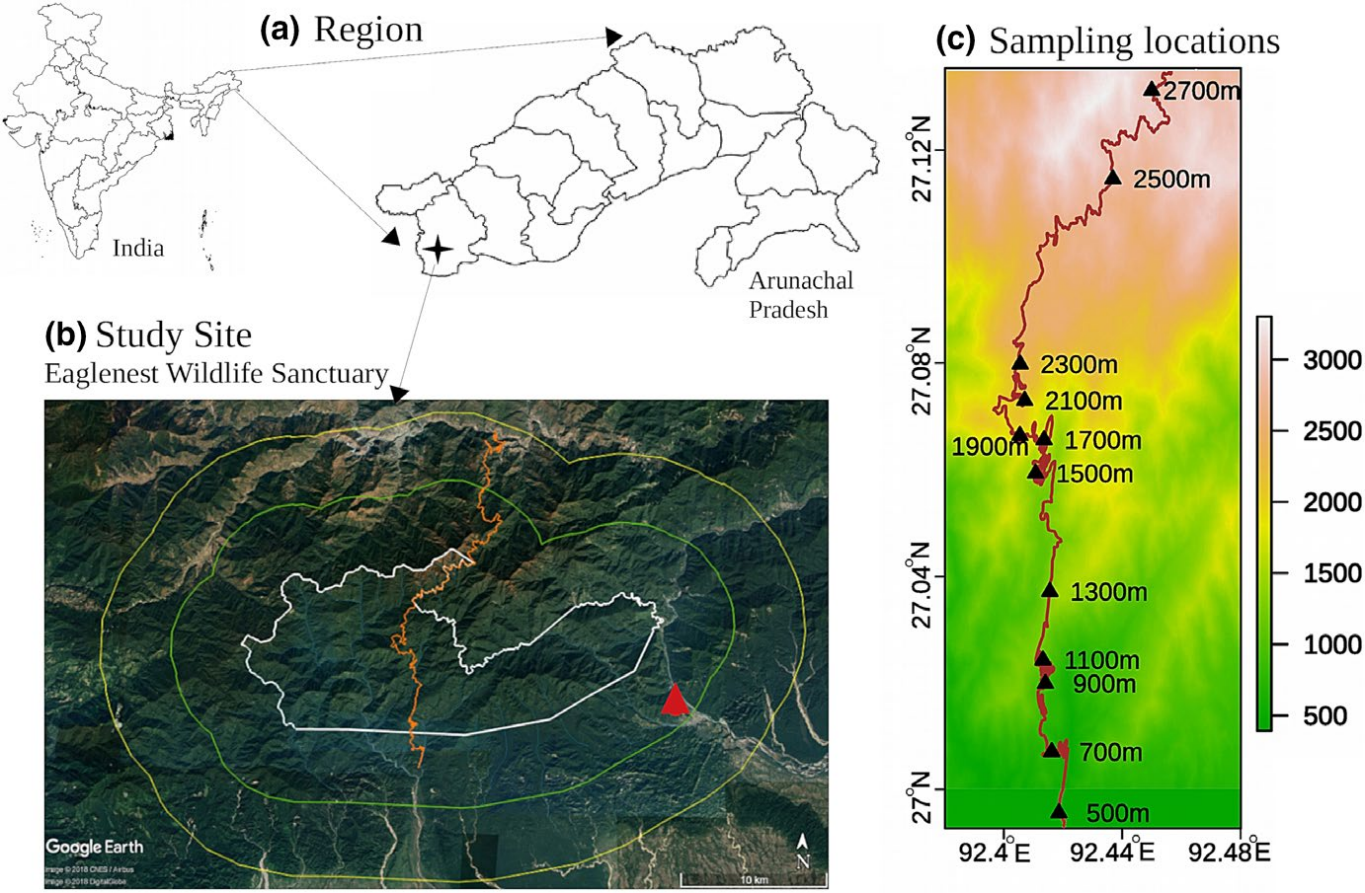
Study site in Eaglenest wildlife sanctuary, India. (a). Location of the study site in West Kameng district, Arunachal Pradesh, northeast India (b). A Google Earth image of Eaglenest Wildlife Sanctuary with its boundary marked in white, and that of its 5km buffer strip in green. The dirt track running through the sanctuary, shown in orange, traverses elevations from 100 m in the south to the Eaglenest pass at 2,780 m and down to 1,200 m to the north. The 200 m sampling location, which is outside the wildlife sanctuary, is marked by a red triangle. (c). Digital elevation map showing the Eaglenest track and the sampling locations between 500 m and 2,700 m

Hawkmoth sampling was carried out in Eaglenest Wildlife Sanctuary (see Athreya, 2006b for a detailed description of the sanctuary), a *Protected Area* of 218 km^2^ located between 27^o^02″ 09′ N and 92^o^18″ 35′ E in the eastern Himalayas of Arunachal Pradesh, northeast India (Figure 1). The large elevational range of 3,150 m coupled with high rainfall (>3,000 mm along the southern slopes) has resulted in diverse habitat types ranging from tropical wet evergreen below 900 m to coniferous temperate forests above 2,700 m (Champion & Seth, 1968). The high diversity of this region, believed to be due to its complex terrain and its location at the confluence of the Oriental and Sino‐Japanese floristic and faunistic zones (Holt et al., 2013), makes it a globally important biodiversity hot spot (Orme et al., 2005).

Point sampling was carried out at UV‐illuminated screens on no‐moon nights along a vehicle track characterized by roadside scrub in close proximity to primary forest (5–20 m away). The sampling was carried out in a single compact transect to reduce the impact of variation in gamma diversity while sampling across widely separated transects (McCain, 2007). The 12 elevations between 500 m and 2,770 m, approximately 200 m apart, were clustered in a small stretch spanning just 15 km. The 200 m location, near the village of Tippi, was separated from its nearest neighbor by about 20 km due to the lack of access to suitable habitat along this road (Figure 1). The sampling was completed during a single breeding season (summer) in 2014, in April at 200 m, and May–July at the other elevations.

We set up portable UV screens (Mungee & Athreya, 2020) between 7 p.m. and midnight during the 7 days before and 3 days after the new moon, when the moon was below the visible horizon during those 5 hr. We sampled at 2–5 elevations simultaneously to achieve some degree of uniformity of weather conditions (which can change drastically from day to day) across the elevational gradient. Hawkmoths which arrived at the screen were photographed unfettered, in their natural posture against the reference grid printed across the entire screen, then captured for marking (by clipping a tiny portion of the forewing apex) to avoid double counting, and for collection of the two middle legs for DNA, and subsequently released.

We aimed to collect similar number of total individuals at each elevation because of the high daily variability observed in hawkmoth numbers at a light screen, even within the 10‐day no‐moon period (Appendix S1: Figure A1). Previous studies have also reported high fluctuations in moth activity due to local weather, temperature, wind, cloud, rains, etc. (Beck et al., 2008; McGeachie, 1989; Schulze & Fiedler, 2003). It has been suggested that the number of individuals is a better measure of the sampling effort for moths than the number of trap nights (Willott, 2001).

### 2.2 Species identification and trait measurement

We assigned individuals to morphospecies using the online resources made available by Kitching and collaborators (http://sphingidae.myspecies.info/, http://tpittaway.tripod.com/china/china.htm; Kitching, 2019). We recorded a total of 4,731 hawkmoth individuals from 13 elevational communities that could be identified to morphospecies; it included 80 morphospecies from 30 genera and all 3 Sphingid subfamilies (Sphinginae, Macroglossinae, and Smerinthiinae). The details are provided in supplementary section A.

We measured body length, thorax width, wing custom length, and wing breadth from field images after calibration and distortion corrections (Mungee & Athreya, 2020). We derived from these primary measurements the three functional traits of body mass, wing loading, and wing aspect ratio. We could measure traits reliably for 3,301 individuals (70% of the identified sample) from 76 morphospecies and 30 genera. The rest either did not sit on the gridded screen, or image analysis showed high error in trait estimation. Supplementary section B provides a brief description of the trait measurement procedure. More details may be obtained from Mungee and Athreya (2020).

Apart from the *Trait data set* of 3,301 individuals mentioned above, we repeated the analyses for two other sets of data to understand the impact of incompleteness: (a) *Diversity data set* of 4,731 individuals: This included another 1,430 individuals identified to morphospecies whose traits could not be measured. We filled in the missing trait data by randomly resampling the traits from others of the same species in the same community. For example, we could measure traits for only 66 of the 79 individuals of the species *Acosmerycoides harterti* at elevation 700 m. The remaining 13 individuals were assigned trait values drawn at random from the set of 66 individuals. (b) *Trait data without E1700*: The moth community from 1,700‐m elevation suffered a disproportionate loss of trait data. Heavy rainfall toward the end of the session just after many moths had arrived at the screen precluded photography against the gridded screen. So we transported the moths individually to a nearby shelter and photographed them for species identification but without trait information.

We assessed the completeness of our samples using taxonomic (of *diversity data set*) and functional trait (of *trait data set*) rarefaction curves using R package *evolqg* (Melo et al., 2015; see Supplementary 1 for details).

### 2.3 Environmental variables

We explored the variation of 4 environmental variables along the elevational transect: mean annual temperature (MAT), mean annual precipitation (APPT), plant productivity (EVI: enhanced vegetation index), and air density (AD). MAT and APPT with a spatial resolution of 1 km^2^ were downloaded from *worldclim* (https://www.worldclim.org/) for the years 2004–2014. EVI was obtained from NASA’s MODIS satellite products (MOD13Q1) with a resolution of 250 m. Temperature and productivity influence body size even in ectotherms via behavioral thermoregulation (Zamora‐Camacho et al., 2014) and resource availability (McNab, 2010). Precipitation was included as a predictor since it is expected to influence productivity. Air density (and temperature) changes the viscosity of air which impacts the flying ability of insects (Hassall, 2015).

Principal component analysis of the 4 variables yielded a first principal component which explained 91% of the variance (Appendix S1: Figure C2), and was strongly positively correlated with elevation (*R* ^2^ = 0.95; *p* < .005; Appendix S1: Figure C3). We considered using the first principal component as a composite environmental variable (e.g., Le Bagousse‐Pinguet et al., 2014; Subedi et al., 2019) but, as explained in the Discussion (while comparing the environmental gradient in different studies), decided that the elevation as an environmental surrogate was the better option. The details of the analysis of the environmental data are provided in Appendix S1: Section C.

### 2.4 Trait variation across the elevational gradient

We used two approaches to examine the response of hawkmoth community‐trait values across the elevational gradient. First, we investigated the change in functional “alpha” diversity across the gradient using the community abundance‐weighted mean trait value (CWM; Lavorel et al., 2008). The CWM for the *k*‐th community was calculated using CWM*_k_* = Σ*a_ik_* *t_ik_* where *a_ik_* is the relative abundance of the *i*‐th species in the *k*‐th community, and *t_ik_* is the mean of all the individuals of the *i*‐th species within the *k*‐th community. The change in community mean with elevation was assessed using ordinary least squares regression. We also calculated the CWM using regional species means: CWM*_k_* = Σ*a_ik_* *t_i_*, where *a_ik_* is the relative abundance of the *i*‐th species in the *k*‐th community, and *t_i_* is the mean value for the *i*‐th species across the entire region (i.e., all communities).

Second, we quantified the change in trait across the gradient using the degree of overlap of the kernel density distributions (area of intersection) for all pairs of communities, that is, essentially the functional “beta” diversity (Mouillot et al., 2005). The kernel density distributions were constructed in a nonparametric manner without assuming an underlying distribution for community‐trait values (Carmona et al., 2016). We used ordinary least square regression to examine the change in overlap for each trait (individually) with increasing elevational distance between the communities.

### 2.5 Internal and external filters influencing community assembly

We employed *T‐statistic* metrics (Violle et al., 2012) to infer the operation of internal and external filters influencing hawkmoth community assembly across the elevational gradient. In the context of this study, the “region” spans the elevational range of 200–2770 m. It consists of 13 elevational “communities” separated from each other by about 200 m. The region hosts many species, and the individuals of a species within a community constitute a population; that is, the populations of different species constitute a community.

Three variance ratios of *T‐statistics* at nested spatial and taxonomic scales were obtained as follows:

#### 2.5.1 Internal filter metric

$T_{\text{IP/IC}}=\left(\sigma_{\text{IP}}^{2}/\sigma_{\text{IC}}^{2}\right)$, the ratio of the variance of trait values within a population (averaged over all species in that community) to that of trait values of all individuals (regardless of species) within the community.

#### 2.5.2 External filter metric using individual trait values

$T_{\text{IC/IR}}=\left(\sigma_{\text{IC}}^{2}/\sigma_{\text{IR}}^{2}\right)$, the ratio of variance of trait values of individuals within a community (regardless of species) to that of all individuals within the entire region.

#### 2.5.3 External filter metric using species mean values

$T_{\text{PC/PR}}=\left(\sigma_{\text{PC}}^{2}/\sigma_{\text{PR}}^{2}\right)$, the ratio of variance of population mean trait values within a community to that of population mean trait values within the regional pool.

The observed metrics were compared to those obtained from the simulated null models (obtained by randomizing the actual data) to detect nonrandom trait structure within and across communities. Details on generation of the null models are provided in Appendix S1: Table D1. The standardized effect size (SES) of the deviation of the observed value from the null model was calculated as follows:

$$\text{SES}=\frac{I_{\text{obs}}‐I_{\text{null}}}{\sigma_{\text{null}}},$$

where *I*_obs_ is the observed value of a metric, and *I*_null_ and *σ*_null_ are the mean and standard deviation of the simulated null model replicates.

Following Neyret et al. (2016), we calculated *T‐statistics* using log‐transformed values of the traits to remove potential scaling effects between the mean value and the standard deviation.

Though *T‐statistic* parameters are closely related to each other, they provide subtly different information. Taking the example of the internal filter metric: $\sigma_{\text{IP}}^{2}$, the intrapopulation variance, is a measure of the average niche width of species. The intracommunity variance, $\sigma_{\text{IC}}^{2}$ (calculated using individual trait values), is a measure of the total niche space occupied by the community, in response to external constraints (filters). Their ratio, which is *T*_IP/IC_, is the niche width of a species relative to that of co‐occurring species in that community; that is, it is a measure of processes which decide species coexistence, of which interspecific competition is an oft‐invoked example (e.g., MacArthur & Levins, 1967). The variation of this metric along environmental gradients has been used in recent years to estimate the change in overall niche width and/or niche‐packing (e.g., Hulshof et al., 2013; Le Bagousse‐Pinguet et al., 2014; Wu et al., 2019). On the other hand, SES of *T*_IP/IC_ estimates the degree of nonrandomness of trait distribution within a community, and hence is a measure of the strength of the internal filter; that is, *T*_IP/IC_ and SES of *T*_IP/IC_ are associated with the same process but are slightly different measures. Apart from testing for the degree of deviation from randomness of the metrics, we also checked their correlation with elevation and species richness following Violle et al. (2012).

*T*_PC/PR_ and *T*_IC/IR_ have been contrasted in the literature in a somewhat confusing language as measuring the operation of external filters at the “level of species” and at the “level of individuals,” whereas in actuality selection and filters operate at the level of individuals. The terminology is meant to highlight the difference in the (statistical) ability to detect external filtering when calculated with and without intraspecific variance.

We also assessed the relationship between the individual metrics on the one hand, and elevation and species richness on the other using ordinary least square regression.

All the analyses were performed in the R programming software; version 3.4.4 (R Development Core Team, 2015) using the following packages: *vegan 2.5.4* for computing species richness, diversity indices, taxonomic rarefaction curves, and environmental variables PCA scores (Oksanen et al., 2007); *evolqg 0.2.6* for functional rarefaction curves (Melo et al., 2015); *FD 1.0.12* for CWM analysis (Laliberté et al., 2014); *sfsmisc 1.1‐3* for the trait kernel density analysis (Maechler et al., 2019); and *cati 0.99.2* (Taudiere & Violle, 2016) for calculating the *T‐statistics and* generating null models.

## 3 RESULTS

The results presented here are for the *Trait data set* (3,301 individuals). The results for the *Diversity* data set are similar and are presented in Supplementary section E. The result for the *Trait set without E1700* was also similar and so has not been shown.

### 3.1 Trait variation across the environmental gradient

**FIGURE 2:**
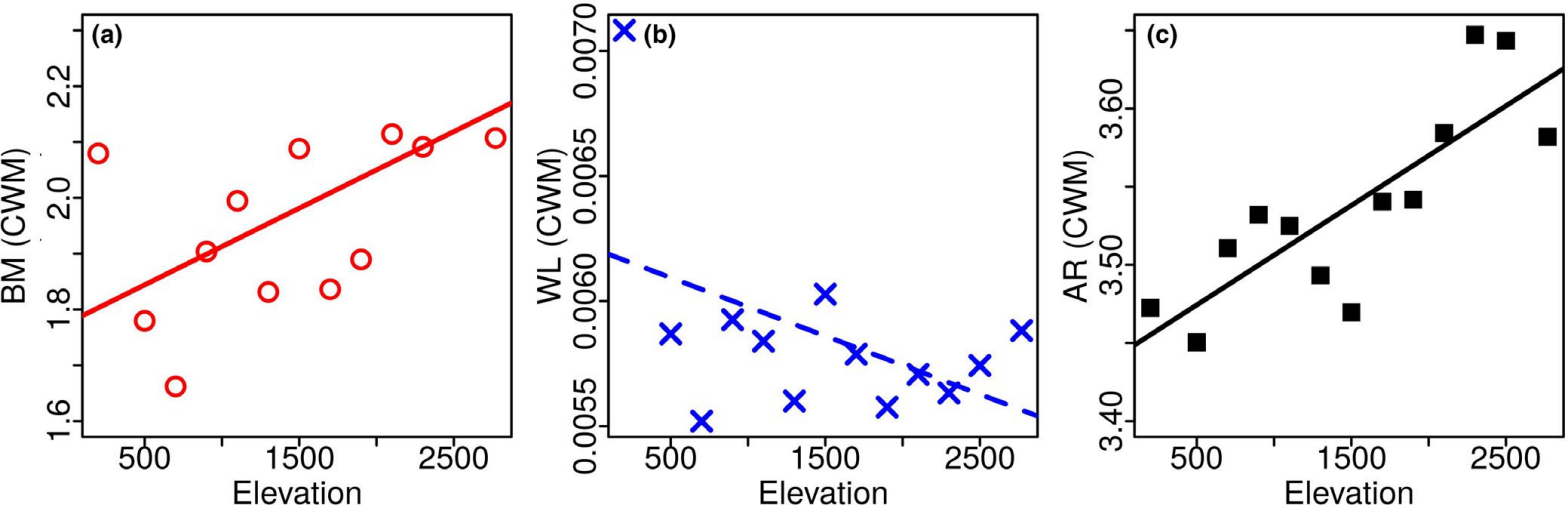
Relationship between hawkmoth community mean trait and elevation. The plots show the change in community‐weighted means of (a). body mass (BM), (b). wing loading (WL), and (c). wing aspect ratio (AR) plotted against elevation. The community mean values were calculated using the population‐specific mean trait for each species in a community. The dashed and solid lines indicate regression fits significant at the 90% (*p* < .1) and 95% (*p* < .05) levels, respectively. The regression parameters are in Table 1

Community‐weighted means of body mass and aspect ratio exhibited a significant positive relationship with elevation (Table 1, Figure 2; body mass: *r^2^* = 0.28, *p* < .05; wing aspect ratio: *r^2^* = 0.64, *p* < .001). The negative relationship between wing loading and elevation was marginally less significant (*r^2^* = 0.10, *p* = .10). There was little difference when CWM was calculated with and without incorporating intraspecific variation. The difference between the slopes for the two cases was not statistically significant (body mass: *Fisher's Z* = 0.43, *p* = .33; wing loading: *Z* = –0.32, *p* = .37; wing aspect ratio *Z* = 0.19, *p* = .42).

**TABLE 1:** Linear regression of hawkmoth community traits with elevation. (a) The community mean trait value was calculated using the population mean trait values weighted by local abundance. (b) The overlap was measured for the trait kernel distributions of pairs of communities and regressed against the elevational separation between them

	Intercept ± *SE*	Slope ± *SE*	Adj. *R* ^2^	*p*
Community mean trait value with elevation				
Body mass	1.78 ± 0.10	(1.37 ± 0.57) x 10^–4^	0.28	**<.05**
Wing loading				
With 200 m	(6.21 ± 0.22) x 10^–3^	(−2.33 ± 1.32) x 10^–7^	0.15	.10
Without 200 m	(5.78 ± 0.12) x 10^–3^	(−1.34 ± 6.75) x 10^–8^	–0.10	.85
Wing aspect ratio	3.44 ± 0.03	(6.37 ± 1.35) x 10^–5^	0.64	**<.005**
Trait distribution overlap with elevational separation				
Body mass	0.88 ± 0.02	(−6.30 ± 1.37) x 10^–5^	0.21	**<.005**
Wing loading	0.91 ± 0.03	(−5.05 ± 2.31) x 10^–5^	0.05	**<.05**
Wing aspect ratio	0.90 ± 0.01	(−4.61 ± 0.89) x 10^–5^	0.25	**<.005**

**FIGURE 3:**
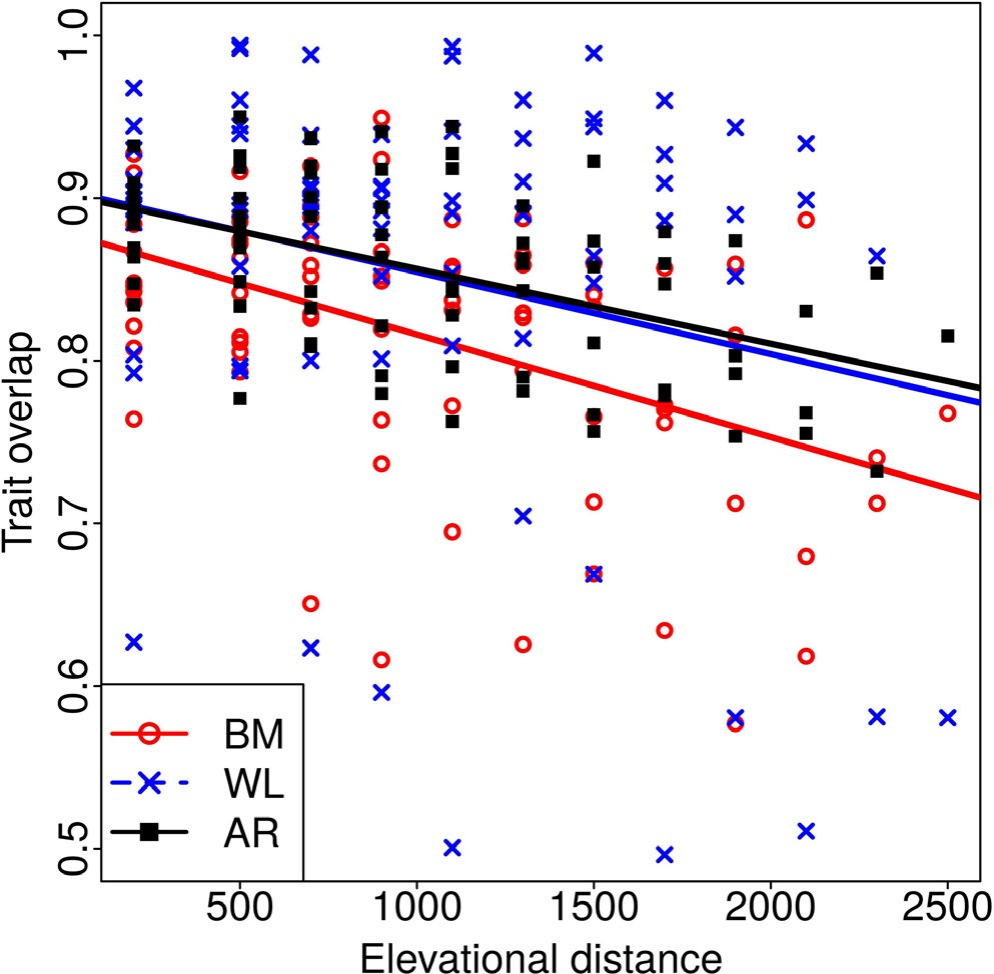
Relationship between hawk moth community‐trait overlap and elevational distance. The plot shows the scatter and the regression lines for the relationship between the overlap in trait distribution functions for pairs of communities and the elevational distance between them. The three traits plotted are body mass (BM), wing loading (WL), and wing aspect ratio (AR). The overlap for a pair of communities was calculated from the area of intersection of their trait kernel density distributions. The solid lines indicate regression fits significant at the 95% (*p* < .05) levels. The regression parameters are in Table 1

The reduction in trait overlap with increasing elevational distance (Figure 3) was significant for all traits (Table 1, Figure 3; body mass: *r^2^* = 0.21, *p* < .005; wing loading: *r^2^* = 0.05, *p* < .05; wing aspect ratio: *r^2^* = 0.25, *p* < .005).

### 3.2 Internal and external filters influencing community assembly

#### 3.2.1 Deviations of communities from randomness

The observed values of the three metrics of *T‐statistics* are listed in Appendix S1: Table D2. The observed SES values of the three *T‐statistic* metrics and the distribution of the same from simulated null models are provided in Appendix S1: Table D3. Traits for which the SES values lie outside the 95 percent range of the null distribution are considered to have a distribution which deviates significantly from randomness.

**FIGURE 4:**
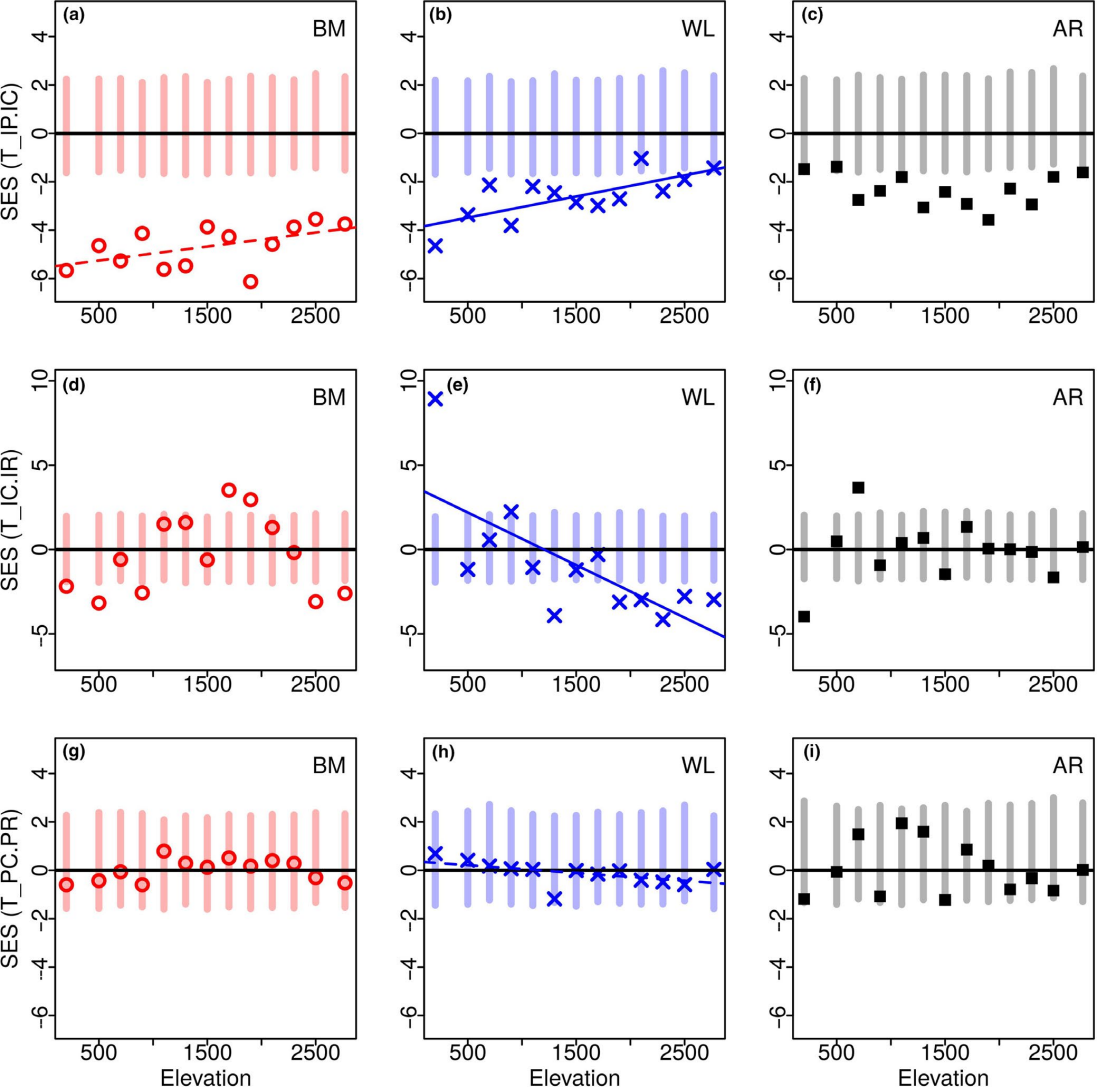
*T‐statistics* of hawkmoth functional traits across an elevation*al* gradient. The plots show the standardized effect sizes (SES) of *T‐statistics* metrics for body mass (BM), wing loading (WL), and wing aspect ratio (AR) for each of the 13 elevational communities. The vertical bars represent the 95% distribution of simulated null communities, and the dots are the observed values. The metrics are variance ratios of (a) *T*_IP/IC_: intrapopulation to intracommunity (b) *T*_IC/IR_: intracommunity to regional, assessed using individual trait values, and (c) *T*_PC/PR_: intracommunity to regional, assessed using population mean values. The dashed and solid lines indicate regression fits significant at the 90% (*p* < .1) and 95% (*p* < .05) levels, respectively. The regression parameters are in Table 2

Figure 4 shows plots of SES for all three *T‐statistic* metrics for all three traits versus elevation. SES values of *T*_IP/IC_ were significantly lower than the null model for all three traits (in all 13 communities for body mass, and in 11 of 13 communities for wing loading and wing aspect ratio; three of the four at the very edge); that is, the dispersion of the trait values of individual species within a community was smaller than the dispersion for the community as a whole, indicating strong internal filtering. SES values of *T*_IC/IR_, an indicator of external filters, were more variable. Values for body mass were significantly lower than null at some communities at both ends of the elevational gradient but higher than null in between. Values for wing loading were significantly lower than null in about half the elevations but lay well above at 200 m. Values of wing aspect ratio were significantly different from null only at 200 m (lower) and 700 m (higher). In all, 12 community‐trait pairs were lower than the null distribution, 5 were higher, and 22 were consistent with the communities being random subsets of the regional pool. SES values of *T*_PC/PR_ were not significantly different from null for any trait‐community combination.

### 3.3 Relationship between T‐statistic metrics and elevation

Both body mass and wing loading showed a trend in which the intrapopulation distribution was increasingly closer to being a random subset of the intracommunity distribution toward higher elevation (two plots in the top row of Figure 4, Table 2). This is reflected in a correlation between elevation and SES values of *T*_IP/IC_ of body mass (*r^2^* = 0.15, *p* = .06) and wing loading (*r^2^* = 0.52, *p* < .005). In the case of wing loading, we also observed a negative correlation between elevation on the one hand and SES values of *T*_IC/IR_ (*r^2^* = 0.47, *p* < .05) and of the related *T*_PC/PR_ (*r^2^* = 0.23, *p* = .05). Interestingly, all the communities in the case *T*_PC/PR_ and half the communities in the case of *T*_IC/IR_ were actually consistent with being random subsets of the regional pool.

**TABLE 2:** Linear regression of *T‐statistic* parameters with elevation

Parameter	Trait	Intercept ± *SE*	Slope ± *SE*	Adj. *R* ^2^	*p*
Intrapopulation variance $\sigma_{\text{IP}}^{2}$	Body mass	(8.56 ± 0.99) x 10^–3^	(−6.16 ± 5.87) x 10^–7^	0.01	.32
	Wing loading	(5.68 ± 0.72) x 10^–3^	(−6.92 ± 4.27) x 10^–7^	0.12	.13
	Wing aspect ratio	(1.36 ± 0.19) x 10^–3^	(−1.70 ± 1.12) x 10^–7^	0.10	.16
Intracommunity variance $\sigma_{\text{IC}}^{2}$	Body mass	(3.47 ± 0.58) x 10^–2^	(−1.35 ± 3.45) x 10^–6^	−0.08	.70
	Wing loading				
	With 200 m	(1.31 ± 0.14) x 10^–2^	(−2.87 ± 0.82) x 10^–6^	0.49	**.004**
	Without 200 m	(1.10 ± 0.01) x 10^–2^	(−1.79 ± 0.68) x 10^–6^	0.35	**.03**
	Wing aspect ratio	(2.11 ± 0.29) x 10^–3^	(−1.17 ± 17.3) x 10^–8^	−0.09	.95
*T*_IP/IC_ ($\sigma_{\text{IIP}}^{2}$/$\sigma_{\text{IIC}}^{2}$)	Body mass	0.257 ± 0.041	(−2.43 ± 2.41) x 10^–5^	0.001	.36
	**Wing loading**	0.427 ± 0.079	(8.67 ± 4.69) x 10^–5^	0.17	**.09**
	**Wing aspect ratio**	0.671 ± 0.077	(−8.76 ± 4.61) x 10^–5^	0.18	**.08**
SES of *T*_IP/IC_	**Body mass**	−5.543 ± 0.459	(0.58 ± 0.27) x 10^–3^	0.22	**.06**
	**Wing loading**	−3.920 ± 0.421	(0.87 ± 0.25) x 10^–3^	0.48	**<.005**
	Wing aspect ratio	−2.070 ± 0.427	(0.18 ± 0.25) x 10^–3^	−0.04	.49
SES of *T*_IC/IR_	Body mass	−0.944 ± 1.458	(0.41 ± 0.87) x 10^–3^	−0.07	.64
	**Wing loading**	3.750 ± 1.543	(−0.31 ± 0.09) x 10^–2^	0.47	**<.05**
	Wing aspect ratio	−0.247 ± 1.12	(0.93 ± 6.65) x 10^–4^	−0.09	.89
SES of *T*_PC/PR_	Body mass	−0.172 ± 0.284	(0.12 ± 0.17) x 10^–3^	−0.04	.49
	**Wing loading**	0.375 ± 0.252	(−0.32 ± 0.15) x 10^–3^	0.23	**.05**
	Wing aspect ratio	0.302 ± 0.698	(−0.17 ± 0.41) x 10^–3^	−0.07	.69

Note

The regressions which are statistically significant with *p* < .1 are in bold font. *T*_IC/IR_ and $\sigma_{\text{IC}}^{2}$ differ only by the factor $\sigma_{\text{IR}}^{2}$, which is a property of the region (value for body mass: 3.704 10^−2^; wing area: 0.996 10^−2^; wing aspect ratio: 0.209 10^−2^) and hence the same for all communities.

**FIGURE 5:**
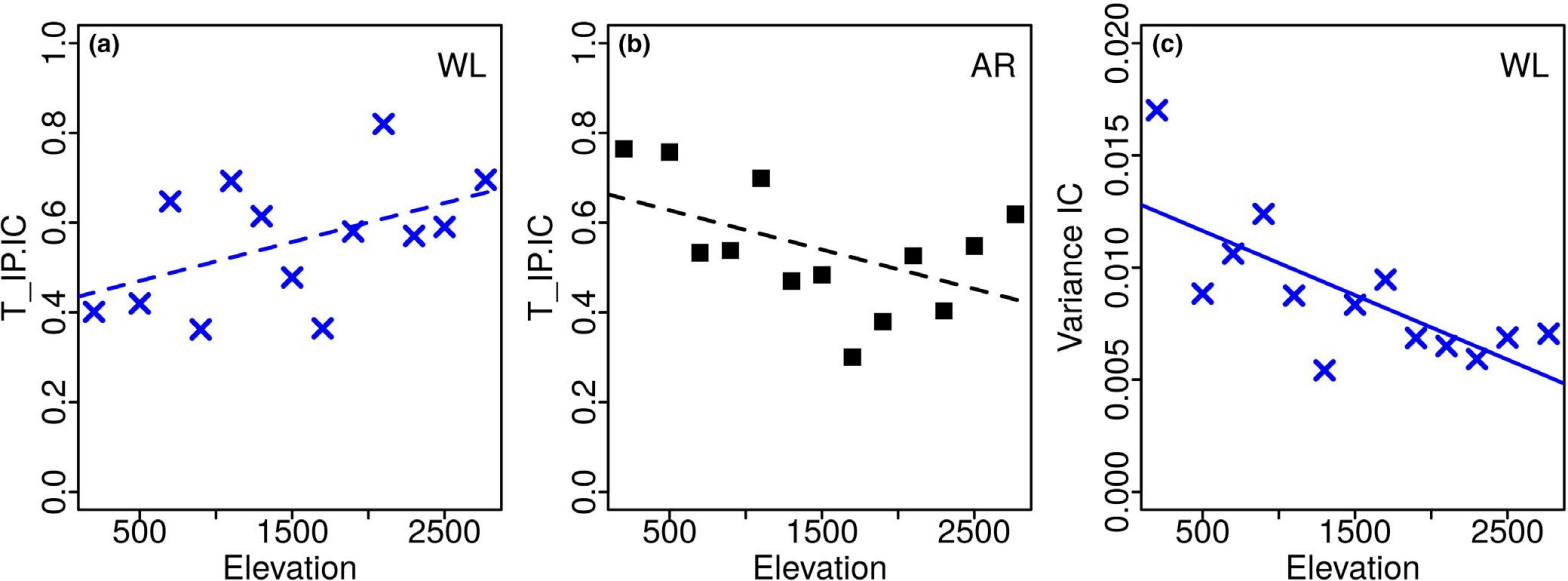
*T‐statistic* parameters of hawkmoth functional traits across an elevational gradient The plots show the *T‐statistic* parameters which have a statistically significant relationship with elevation: (a) intrapopulation to intracommunity variance ratio (*T*_IP/IC_) of wing loading, (b) intrapopulation to intracommunity variance ratio (*T*_IP/IC_) of wing aspect ratio, and (c) intracommunity variance ($\sigma_{\text{IC}}^{2}$) of wing loading. The dashed and solid lines indicate regression fits significant at the 90% (*p* < .1) and 95% (*p* < .05) levels, respectively. The regression parameters are in Table 2

The regression results for the elevational dependence of intrapopulation variance ($\sigma_{\text{IP}}^{2}$), intracommunity variance ($\sigma_{\text{IC}}^{2}$), and the internal filter metric (*T*_IP/IC_) are shown in Figure 5 and Table 2. It should be noted that *T*_IP/IC_ is the ratio of the other two quantities, having intracommunity variance in the denominator. Body mass showed no significant relationship with elevation in any of the three parameters (*r^2^* < 0.01; *p* = .32 to 0.7). Wing loading showed a significant relationship for intracommunity variance (*r^2^* < 0.49; *p* = .004) and a marginal relationship for *T*_IP/IC_ (*r^2^* < 0.17; *p* = .09) but none for intrapopulation variance (*r^2^* < 0.12; *p* = .13). Wing aspect ratio showed a marginal relationship for *T*_IP/IC_ (*r^2^* < 0.18; *p* = .08) but none for intrapopulation and intracommunity variances (*r^2^* ≤ 0.1; *p* = .16–0.95).

### 3.4 Relationship between T‐statistic metrics and species richness

**FIGURE 6:**
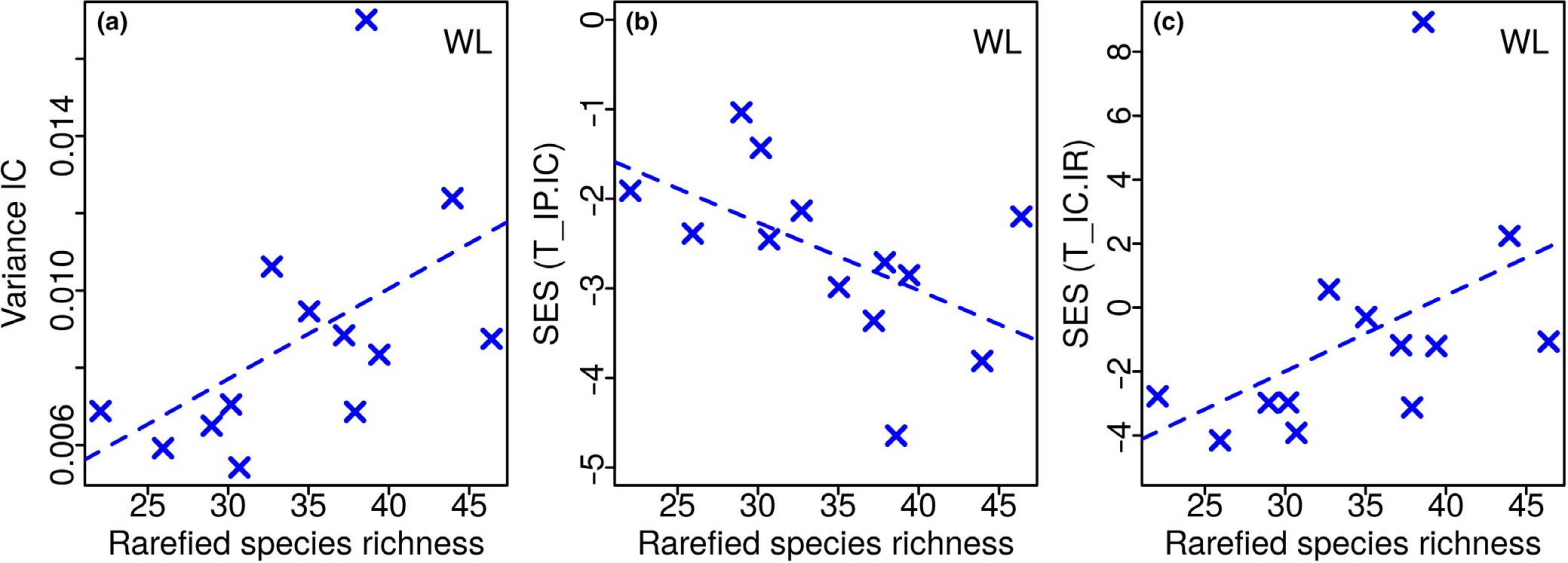
*T‐statistic* parameters of hawkmoth functional traits across a species richness gradients. The plots show the *T‐statistic* metrics which have a statistically significant relationship with species richness: (a) intracommunity variance ($\sigma_{\text{IC}}^{2}$) of wing loading, (b) SES of intrapopulation to intracommunity variance ratio (*T*_IP/IC_) of wing loading, and (c) SES of intracommunity to regional variance ratio (*T*_IC/IR_) of wing loading. The dashed and solid lines indicate regression fits significant at the 90% (*p* < .1) and 95% (*p* < .05) levels, respectively. The regression parameters are in Table 3

The regression results for the species richness dependence of *T‐statistic* metrics are listed in Table 3. The statistically significant relationships, all associated with wing loading, are plotted in Figure 6: intrapopulation variance $\sigma_{\text{IP}}^{2}$ (*r^2^* = 0.20, *p* = .07), intracommunity variance $\sigma_{\text{IC}}^{2}$ (*r^2^* = 0.24, *p* = .05), and *T*_IP/IC_ (*r^2^* = 0.16, *p* = .10).

**TABLE 3:** Linear regression of *T‐statistic* parameters with species richness

Parameters	Trait	Intercept ± *SE*	Slope ± *SE*	Adj. *R* ^2^	*p*
Intrapopulation variance $\sigma_{\text{IP}}^{2}$	Body mass	(5.38 ± 2.39) x 10^–3^	(6.53 ± 6.79) x 10^–5^	−0.01	.36
	Wing loading	(2.87 ± 1.84) x 10^–3^	(5.13 ± 5.23) x 10^–5^	−0.003	.35
	Wing aspect ratio	(0.78 ± 0.49) x 10^–3^	(0.93 ± 1.38) x 10^–5^	−0.05	.52
Intracommunity variance $\sigma_{\text{IC}}^{2}$	Body mass	(3.16 ± 1.39) x 10^–2^	(1.46 ± 3.96) x 10^–4^	−0.08	.72
	**Wing loading**	(0.69 ± 4.10) x 10^–3^	(2.34 ± 1.17) x 10^–4^	0.20	**.07**
	Wing aspect ratio	(2.27 ± 0.70) x 10^–3^	−(0.54 ± 1.98) x 10^–5^	−0.08	.79
*T*_IP/IC_	Body mass	0.17 ± 0.10	(1.43 ± 2.86) x 10^–3^	−0.07	.63
	Wing loading	0.83 ± 0.20	−(7.88 ± 5.68) x 10^–3^	0.07	.19
	Wing aspect ratio	0.33 ± 0.20	(6.02 ± 5.82) x 10^–3^	0.01	.32
SES of *T*_IP/IC_	Body mass	−2.78 ± 1.17	−(5.49 ± 3.32) x 10^–2^	0.13	.13
	**Wing loading**	0.01 ± 1.23	−(7.59 ± 3.48) x 10^–2^	0.24	**.05**
	Wing aspect ratio	−2.67 ± 1.04	(0.97 ± 2.96) x 10^–2^	−0.08	.75
SES of *T*_IC/IR_	Body mass	−1.75 ± 3.50	(4.13 ± 9.96) x 10^–2^	−0.07	.69
	**Wing loading**	−9.12 ± 4.66	(2.37 ± 1.32) x 10^–1^	0.16	**.10**
	Wing aspect ratio	0.69 ± 2.67	−(2.31 ± 7.60) x 10^–2^	−0.08	.77

Note

Species richness was calculated from the rarefaction curves. The regressions which are statistically significant with *p* < .1 are in bold font. *T*_IC/IR_ and $\sigma_{\text{IC}}^{2}$ differ only by the factor$\sigma_{\text{IR}}^{2}$, which is a property of the region (value for body mass: 3.704 10^−2^; wing area: 0.996 10^−2^; wing aspect ratio: 0.209 10^−2^) and hence the same for all communities.

## 4 DISCUSSION

We investigated aspects of community assembly of hawkmoths at 13 elevations across a 200–2,770 m elevational gradient in the eastern Himalayas. Specifically, we evaluated the role of internal and external filters in deciding the composition of local communities derived from the regional species pool. We measured body mass, wing loading, and wing aspect ratio of 3,301 hawkmoth individuals from 76 species to evaluate the variation in community‐trait metrics across this elevational gradient. We first showed that the three traits are indeed “functional” response traits from their significant variation across the elevational gradient. We found strong support for the role of internal filters for each of the three traits in all communities using the corresponding *T‐statistic* metric *T*_IP/IC_ of Violle et al. (2012). The metric *T*_IC/IR_, which uses individual trait values, was less emphatic in its support for the role of external filters in community assembly. The corresponding metric for external filters using species mean trait values, *T*_PC/PR_, was not significantly different from the null expectation of no external filter. However, the role of external filters was evident from the change in community mean values of the three traits. Finally, we showed that the presence of external filters may also be inferred from the directional change in any metric across the elevational range, including the standardized effect size (SES) of *T*_IP/IC_; hitherto, this metric has been used only as an indicator of internal filters.

The eastern Himalayas are among the most important and yet the least studied, of global biodiversity hot spots. We did not come across any previous systematic collection of individual‐level trait data for any faunal group from the region. Our intensive sampling effort in a single compact region during a single season yielded 80 hawkmoth species. In comparison, the checklist of hawkmoths for all of India is only a factor three higher (Kitching et al., 2014). Similarly, hawkmoth checklists of many countries in neighboring South‐East Asia consist of 100–160 species (Beck & Kitching, 2009), suggesting that we have achieved a good degree of completeness in sampling the hawkmoth community. We also confirmed the adequacy of the sampling effort using rarefaction curves for both species (Appendix S1: Figure A2) and traits (Appendix S1: Figure A4).

### 4.1 Environmental gradient

Identifying the most important environmental factor and its mechanistic role in community assembly is a difficult exercise. Of the previous studies using *T‐statistics*, three used nonparametric environmental classes (Danet et al., 2018; Khalil et al., 2019; Neyret et al., 2016), five used a surrogate (latitude: Hulshof et al., 2013; Outreman et al., 2018; elevation: Hulshof et al., 2013; Luo et al., 2016; Neyret et al., 2016; Wu et al., 2019), one used precipitation and anthropogenic disturbance (Zorger et al., 2019), two dealt with multiple variables (Le Bagousse‐Pinguet et al., 2014; Subedi et al., 2019), and two did not have an obvious gradient (Gusmão et al., 2020; Xavier‐Jordani et al., 2019). Even when the gradient is obvious, teasing apart the confounding factors can be difficult. The three elevational gradients that Hulshof et al. (2013) studied at 3 latitudes are complicated by confounding factors such as species composition (broad‐leaved v/s conifers) and location (proximity to the sea; tropics *v/s* temperate*)*. Furthermore, the terms low‐ and high‐elevation are very contextual, with 2,600 m in south‐west China termed low (Luo et al., 2016) and 1,111 m in Costa Rica labeled high (Hulshof et al., 2013). We suggest that elevational gradients which span both “tropical” and “temperate” regimes (e.g., Neyret et al., 2016; Wu et al., 2019) offer the best opportunities for understanding the impact of environment in community assembly.

In our study temperature, precipitation, air density, and primary productivity, all of which can affect moth body mass and wing dimension, changed along the elevational gradient. We note that our elevational range corresponds to a mean annual temperature change of 10–24°C, or an equivalent latitudinal change of 20°, or 2,200 km. The habitats range from wet tropical forests below 1,000 m to temperate broad‐leaved forests of birch and rhododendron at 2,770 m. Our 13 sampling locations were all in a compact region (less than 20 km), spaced about 200 m in elevation, and on slopes facing the monsoon winds. Therefore, environmental gradient was substantially large, smoothly varying, and regularly sampled.

Some authors have used the principal component analysis to define a composite environmental variable when dealing with multiple variables (Le Bagousse‐Pinguet et al., 2014; Subedi et al., 2019). While this has the advantage of utilizing all measured variables, there is no obvious way of quantifying the role of this artificial variable in any ecological process. Furthermore, since its construction is entirely phenomenological, the composite variable will be unique to each study, precluding both comparison of results and combining data across studies. Alternatively, one can simply use the surrogate itself, especially if it is highly correlated with the composite—the elevation in our case. In its favor, elevation is a well‐defined quantity for comparing results across studies and one which can be used to average data in a meta‐analysis.

### 4.2 Trait variation across the elevational gradient

Body mass and wing aspect ratio showed a significant change in the community mean value along the elevational gradient. The regression of community mean of wing loading was marginally significant at *p* = .1 but fell well below the threshold without the 200 m data point. However, trait overlap between pairs of communities (effectively functional “beta” diversity) decreased with increasing elevational distance between them for all three traits. These results indicate that hawkmoth body mass, wing loading, and wing aspect ratio are indeed responding to the continuously varying environmental gradient. Therefore, these traits qualify as “functional response traits” (Funk et al., 2017; Suding & Goldstein, 2008; Weiher & Keddy, 1995). Many studies have demonstrated a correspondence between species morphological traits (morphospace) and their “performance” or functional strategies (Dehling et al., 2016; Pigot et al., 2016; Price et al., 2014). For instance, Pigot et al. (2016) found that key dimensions of the ecological niche in passerines, including diet, foraging maneuver, and foraging substrate were, to varying extents, predictable on the basis of morphological traits. Ecogeographic studies, which investigate the change of trait values along an environmental gradient (e.g., Bergmann's rule), have a long history. We will be presenting the results of a more detailed study of elevational patterns of body mass, wing loading, and wing aspect ratio in a different publication. In this paper, the elevational patterns of these traits only serve the limited purpose of demonstrating that they are indeed functional response traits.

Interestingly, the only other study of moth community assembly that we encountered used “image complexity” as a trait (Wu et al., 2019). They characterized the color patterns on moth specimen images using a vector with 2,048 dimensions. They then collapse all these dimensions into a single measure of “distance of pattern complexity” between specimens. As the authors themselves admit, it is not clear what this single “trait” represents or what selection pressure this may be responding to.

### 4.3 Community assembly

The realized and fundamental niches of co‐occurring species are key to understanding how local communities are assembled from a “regional” species pool (Kraft et al., 2008). We principally relied on *T‐statistic* metrics to investigate the role of internal and external filters in community assembly.

### 4.4 Internal filters

In our study, 35 out of the 39 trait‐community combinations showed strong internal filtering with another 3 being marginally so (Figure 4, top row). This strong signature of internal filtering is consistent with the results from all studies using *T‐statistics* (cited throughout this paper). However, TIP/IC was not correlated with species richness (Table 3), suggesting a neutral process of community assembly (Clark, 2010; Clark et al., 2010), which at first sight contradicts the nonrandomness of the community. The mean values of *T*_IP/IC_ (i.e., average variance ratios of within‐species to across‐community) are 0.22 for body mass, 0.56 for wing loading, and 0.54 for wing aspect ratio. That is, the average standard deviation ratios of within‐species to across‐community are 47%, 75%, and 73%, respectively. These are not small fractions; that is, most species occupy a large fraction of the community‐trait space, recalling the prediction of neutral theory. Values of *T*_IP/IC_ in previous studies, where they have been reported, are also in the range 0.2–0.6 (e.g., Hulshof et al., 2013; Luo et al., 2016). Of course, the niche of any species is multidimensional and the fractional occupancy in this hypervolume would be the product of the fractional occupancies along all trait dimensions. So, even while it seems that any single species occupies a large fraction of the available space along any single trait axis, it is likely that they segregate quite well in the niche hypervolume. Clearly, combined analysis of multiple traits is indicated. We draw attention to the ability of *T*_IP/IC_ to detect nonrandomness in intraspecific *vis‐a‐vis* intracommunity‐trait structure even when individual species occupy up to 75% of the community‐trait space.

### 4.5 External filters

More than half of the trait‐community combinations were consistent with the communities being random subsets of the regional pool (using *T*_IC/IR_; Figure 4, middle row). Previous studies have also reported that *T*_IC/IR_ does not provide consistent evidence for external filtering across an environmental gradient. The metric *T*_PC/PR_, which measures external filtering while ignoring intraspecific variance, showed an even lower degree of nonrandomness than *T*_IC/IR_ (Figure 4, bottom row). This is consistent with previous results which have highlighted the importance of using intraspecific variance while studying community assembly (e.g., Albert et al., 2011; Bolnick et al., 2011; Cianciaruso et al., 2009; Enquist et al., 2015; Hulshof et al., 2013; Jung et al., 2010; Paine et al., 2011).

*Any* directional variation of *any* trait quantity (mean, variance, or any other metric) across an environmental gradient is a sign of an external filter (HilleRisLambers et al., 2012; Weiher & Keddy, 1995). Therefore, the evidence for demonstrating that the three traits are indeed functional (Figures 2 and 3) will also serve as evidence for an external filter.

The strength of the internal filter (SES of *T*_IP/IC_) changed across the elevational gradient in our study. Body mass and wing loading showed a significant linear pattern with elevation while wing aspect ratio showed a mid‐elevation trough. However, in the absence of a theoretical justification for fitting higher order polynomials, we have refrained from interpreting this wing aspect ratio pattern. Ironically, this variation of the internal filter across the environmental gradient, as with any other trait metric, is also evidence for the action of an external filter. Such a variation has only been reported previously by Zorger et al. (2019). We suggest that this pattern was discernible in this study because the environmental range was large (spanning both tropical and temperate biomes), continuous, and closely sampled (every 200 m).

The decrease in structuring from lower to higher elevations has been previously linked to higher species diversity, and hence competition, at lower elevations (Callaway, 1998; Spasojevic & Suding, 2012; Wang et al., 2008). However, only wing loading (community variance, SES of *T*_IP/IC_, and SES of *T*_IC/IR_) showed a significant correlation with species richness (Table 3; Figure 6).

Curiously, in the case of body mass, while the degree of randomness of *T*_IP/IC_ (SES of *T*_IP/IC_) showed a significant change with elevation, none of its constituents (*σ*_IP_, *σ*_IC_, or even their ratio *T*_IP/IC_) showed such a relationship (Table 2). We note that variances and means are only the simplest parameters of a distribution (of traits), and distributions having the same mean and variance can be very different from each other (e.g., a normal and a uniform distribution). The test for randomness takes into account the details of the distribution of values rather than just their mean and variance. Conversely, even though the SES of *T*_PC/PR_ of body mass and wing loading lay well within the null model envelopes, they exhibited a definite pattern (linear or otherwise) with elevation (Figure 4, bottom row). This has also been observed by Zorger et al. (2019). Therefore, the action of an external filter can be discerned in two different ways: (i) the usual one of communities being nonrandom subsets of the regional pool, and (ii) a directional variation of *any* metric along the environmental gradient. Further, the different quantities that constitute a *T‐statistic* metric (e.g., $\sigma_{\text{IP}}^{2}$, *σ*_IC_, their ratio *T*_IP/IC_, and SES of *T*_IP/IC_) do not always correlate the same way with other variables (e.g., elevation or species richness). Perhaps, these metrics carry more information than hitherto envisaged but interpreting them requires more simulations and carefully designed field studies.

### 4.6 Community variance of traits with elevation

The community variance of wing loading showed a significant reduction with elevation as we had hypothesized (Figure 5, Table 2), but not of body mass and wing aspect ratio. Wing loading determines the efficiency and ease of flight and therefore is a key ecological trait governing mobility for foraging, predator avoidance, finding mates, and dispersal (Alerstam et al., 2007; Nachtigall, 1985; Norberg, 1985; Pennycuick, 1971). Correlations between flight capacity and latitude or elevation have been documented in several species at intra‐ and interspecific levels (Hassall, 2015; Rohner et al., 2015, 2018), but seldom at the community level (Classen et al., 2017; Brehm et al. 2019).The reduction in variance with elevation is consistent with higher environmental selection/filtering on wing loading and may indicate the importance of associated functions such as dispersal in the search for resources in a difficult and patchy environment.

It is not surprising that the change in community variance with elevation is trait specific since the intensity of selection along a gradient should differ between traits. Indeed, Classen et al. (2017) reported opposite trends for intraspecific and interspecific variance of some traits with elevation in honey bees. They explained this in terms of two conflicting considerations: a physiological requirement which favors increasing body size with reducing temperature (or Bergmann's rule; see, e.g., Blackburn et al., 1999; Van Voorhies, 1996) and species‐energy theories which selects for reduction in body mass with elevation (e.g., Brown & Maurer, 1989; Rodríguez et al., 2008). Translating these intra‐ and interspecific results to predict the result at the community level requires a more carefully structured study which is beyond the scope of this work.

Any study such as this necessarily can only deal with a very limited subset of the diversity of an area. Hawkmoths are likely to be in competition with not only moths of other families but also other herbivores (insects and others) in the ecosystem. Internal filters, to which interspecific competition is a likely contributor, have been observed to play a significant role in this and other studies of many taxa. Whether or not a similar study which includes several faunal groups will reach the same conclusion is an open question. The addition of other taxa into this mix can only increase the already high overlap in species trait values within a community (discussed earlier).

Collection and preservation of museum specimens, though useful in many ways, can add a large financial cost to a study of traits. In this study, we accurately measured the traits of free‐ranging moths without collecting them or even constraining them in any manner. This strategy lends itself to a logistically simple and inexpensive way of compiling large multiepoch trait databases to understand how faunal populations are responding to a changing environment, whether due to global climate change or land‐use pattern change in anthropogenic origin.

In conclusion, we have shown that both internal and external filters have influenced the assembly of the hawkmoth community in the eastern Himalayas. The *T‐statistic* metrics that we used have many subtle aspects (like the difference between *T*_IC/IR_ and SES of *T*_IC/IR_) which may provide more insights into community assembly. An examination of previous studies suggests that *T*_IP/IC_ is a sensitive diagnostic of intracommunity‐trait structure, and hence niche complementarity; this is despite each species occupying 50%–75% of the overall community‐trait space. Multitrait *T‐statistics* is likely to bring out a much stronger signal of niche complementarity; developing techniques for combined analysis of multiple traits would be the next step. Combined analysis of multiple taxa which are functionally similar (e.g., all moth families, or even other insect herbivores) provides another open line of enquiry. The *T‐statistic* metric for external filters, when used in the prescribed manner, appears to be less sensitive. However, we inferred the presence of external filtering by examining the directional variation of traits and metrics (including, ironically, the internal filter metric) across the environmental gradient. This was possible because our environmental gradient was large, smoothly varying, well sampled, and quantitative (not just categorical). Finally, this study developed a technique to measure body and wing dimensions of free‐ranging moths. With this technique, one can generate large databases of hundreds of thousands of individuals at relatively little expense, without having to gather and manage a large specimen collection. Body and wing dimensions play an important role in many physiological and ecological processes in moths. With their high species diversity, abundance, ease of sampling, and key role as herbivores in ecosystems, moths are excellent targets for community assembly studies. They are especially suited for studies which require multiepoch and multilocation sampling like ecosystem stability and impact of environmental change on faunal populations.

## CONFLICT OF INTEREST

The authors declare that they have no conflict of interest.

## AUTHOR CONTRIBUTION

**Mansi Mungee:** Conceptualization (equal); Data curation (supporting); Formal analysis (lead); Investigation (equal); Methodology (lead); Project administration (equal); Software (equal); Writing‐original draft (equal); Writing‐review & editing (supporting). **Ramana Athreya:** Conceptualization (equal); Data curation (lead); Formal analysis (equal); Funding acquisition (lead); Investigation (equal); Methodology (equal); Resources (lead); Software (equal); Supervision (lead); Validation (lead); Visualization (lead); Writing‐original draft (equal); Writing‐review & editing (lead).

## Supporting information

Appendix S1

## Data Availability

Species trait data and environmental variables are made available via the online Appendix S1.
