# Supplementary material for: Intraspecific trait variability and community assembly in hawkmoths (Lepidoptera: Sphingidae) across an elevational gradient in the eastern Himalayas, India
Source: Ecol Evol. 2021 Feb 25;11(6):2471–87. doi: 10.1002/ece3.7054 (PMC7981230; doi:10.1002/ece3.7054)
Supplement: Supplementary file 1 — Appendix S1 [file ECE3-11-2471-s001.docx]

**Supplementary Material**

**Intraspecific trait variability and community-assembly of hawkmoths (Lepidoptera: *Sphingidae*) across an elevational transect in the eastern Himalayas, India**

**Mansi Mungee** and **Ramana Athreya**

**A: Hawkmoth Diversity**


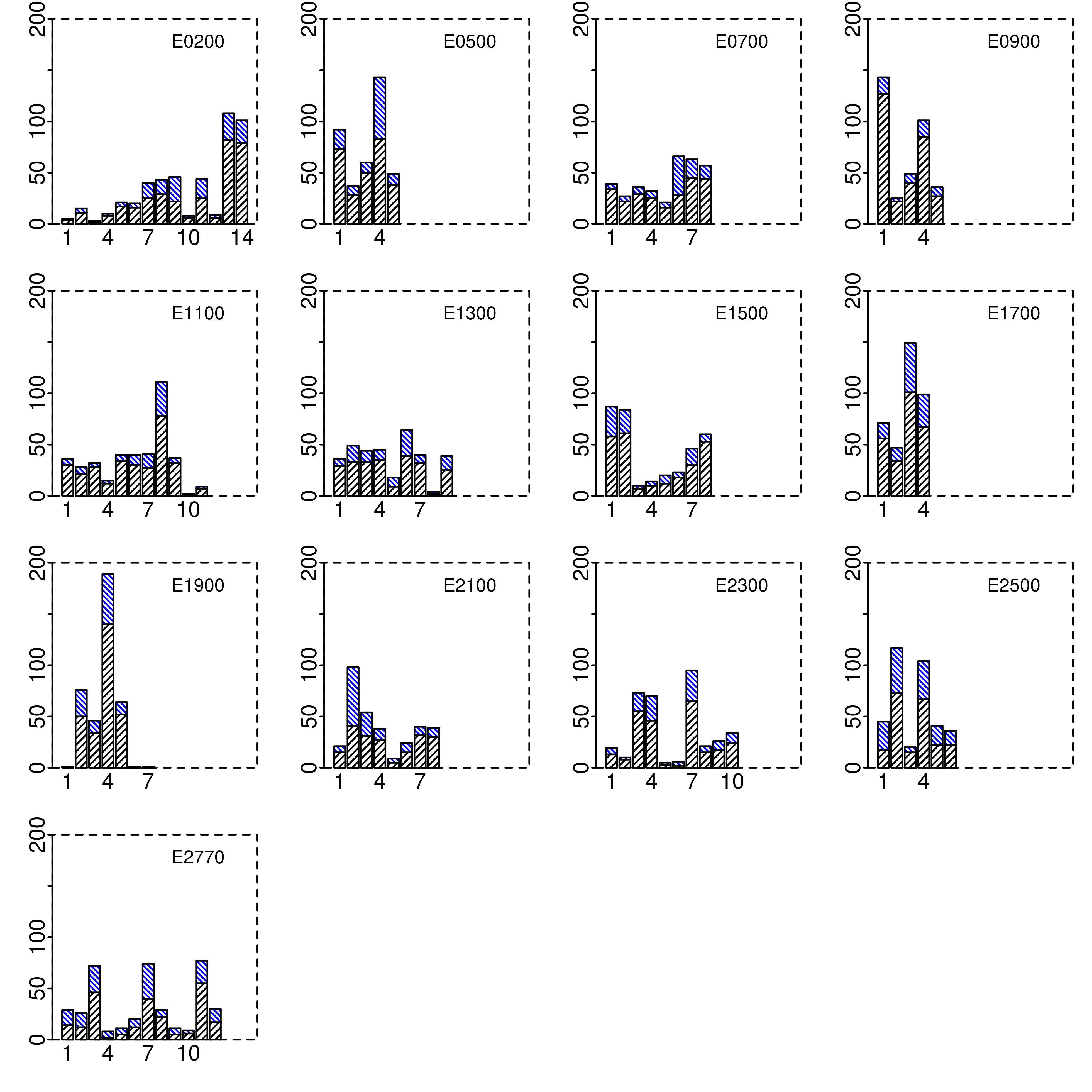
**Figure A1: Nightly hawkmoth visitation rates at light screens.**

The X-axis is the sampling day sequence number at that elevation, and the Y-axis represents the number of hawkmoths. The elevational community is identified by the label in the top-right corner. The blue shaded region represents the number of individuals of the most abundant species on that day.

**Table A1: Elevation-wise sampling data for hawkmoths**

Community: Name of the sampling location (approximate elevation in meters with a “E” prefix for Elevation. N_NIGHT_: Number of trap nights at each corresponding elevation; N_DIV_: Number of individual hawkmoths in diversity data set, i.e. in the total number of individuals at that elevation; N_TDS_: Number of individual hawkmoths in trait data set, i.e. in the subset of individuals from that elevation for which traits were obtained; S_DIV_: Observed species richness in the diversity data set; S_TRAIT_: Observed species richness in the trait data set; R_DIV_: Rarefied species richness in the diversity data set; R_TRAIT_: Rarefied species richness in the trait data; F_DIV_: Diversity using Fisher’s alpha in the diversity data set; F_TRAIT_: Diversity using Fisher’s alpha in the trait data set.

| **Community** | **N_NIGHT_** | **N_DIV_** | **N_TRAIT_** | **S_DIV_** | **S_TRAIT_** | **R_DIV_** | **R_TRAIT_** | **F_DIV_** | **F_TRAIT_** |
| --- | --- | --- | --- | --- | --- | --- | --- | --- | --- |
| **E0200** | 14 | 473 | 429 | 43 | 41 | 39 | 26 | 11.5 | 11.2 |
| **E0500** | 5 | 378 | 298 | 39 | 36 | 37 | 25 | 10.9 | 10.7 |
| **E0700** | 8 | 335 | 271 | 33 | 27 | 33 | 21 | 09.1 | 07.5 |
| **E0900** | 5 | 347 | 224 | 45 | 37 | 44 | 29 | 13.8 | 12.6 |
| **E1100** | 11 | 350 | 248 | 48 | 40 | 46 | 29 | 15.1 | 13.5 |
| **E1300** | 9 | 332 | 208 | 31 | 25 | 31 | 20 | 08.4 | 07.4 |
| **E1500** | 8 | 340 | 173 | 40 | 34 | 39 | 28 | 11.8 | 12.7 |
| **E1700** | 4 | 359 | 103 | 36 | 24 | 35 | 24 | 10.0 | 09.8 |
| **E1900** | 7 | 376 | 296 | 40 | 35 | 38 | 24 | 11.3 | 10.3 |
| **E2100** | 8 | 323 | 207 | 29 | 27 | 29 | 21 | 07.7 | 08.3 |
| **E2300** | 10 | 359 | 299 | 27 | 25 | 26 | 17 | 06.8 | 06.5 |
| **E2500** | 6 | 363 | 249 | 23 | 20 | 22 | 14 | 05.5 | 05.1 |
| **E2770** | 12 | 396 | 296 | 32 | 31 | 30 | 20 | 08.2 | 08.7 |

**Table A2: Summary of the hawkmoth sample diversity**

| Total number of images recorded | 4808 |
| --- | --- |
| No. of images with species level id (diversity data set) | 4731 |
| No. of species in the diversity data | 80 |
| No. of genera in the diversity data | 30 |
| Moths not on reference grids | 1385 |
| No.. of images processed for calibration | 3346 |
| No. of images removed due to poor dedistortion | 20 |
| No. of individuals removed due to trait outlier flags | 25 |
| Total number of individuals used in all trait analysis (trait data set) | 3301 |
| No. of species in the trait data | 76 |
| No. of genera in the trait data | 30 |

**Table A3: Species trait data summary**

The columns are species mean and standard deviation (SD) of body mass, wing loading (x10^-2^) Aspect Ratio, N_DIV_: number of individuals in diversity data set, R_DIV_: relative abundance in diversity data set, and N_TRAIT_: number of individuals in trait data set.

|  | **Body mass** | | **Wing Load (x10^-2^)** | | **Aspect Ratio** | | **N_DIV_** | **R_DIV_** | **N_TRAIT_** |
| --- | --- | --- | --- | --- | --- | --- | --- | --- | --- |
| **Taxon** | **Mean** | **SD** | **Mean** | **SD** | **Mean** | **SD** |  |  |  |
| *Macroglossinae_Acosmerycoides_harterti* | 1.20 | 0.25 | 0.52 | 0.09 | 3.90 | 0.34 | 117 | 2.47 | 101 |
| *Macroglossinae_Acosmeryx_anceus* | 1.30 | 0.26 | 0.61 | 0.11 | 3.31 | 0.22 | 20 | 0.42 | 17 |
| *Macroglossinae_Acosmeryx_naga* | 2.56 | 0.49 | 0.63 | 0.10 | 3.45 | 0.26 | 403 | 8.52 | 259 |
| *Macroglossinae_Acosmeryx_omissa* | 1.88 | 0.38 | 0.62 | 0.10 | 3.23 | 0.19 | 237 | 5.01 | 147 |
| *Macroglossinae_Acosmeryx_sericeus* | 2.12 | 0.51 | 0.67 | 0.13 | 3.25 | 0.21 | 113 | 2.39 | 94 |
| *Macroglossinae_Acosmeryx_shervillii* | 1.98 | 0.61 | 0.62 | 0.14 | 3.28 | 0.26 | 229 | 4.84 | 175 |
| *Macroglossinae_Acosmeryx_sinjaevi* | 1.92 | 0.53 | 0.63 | 0.17 | 3.28 | 0.17 | 16 | 0.34 | 11 |
| *Macroglossinae_Ampelophaga_species* | NA | NA | NA | NA | NA | NA | 2 | 0.01 | 0 |
| *Macroglossinae_Ampelophaga_dolichoides* | 1.27 | 0.23 | 0.48 | 0.06 | 3.50 | 0.25 | 67 | 1.42 | 57 |
| *Macroglossinae_Ampelophaga_khasiana* | 1.66 | 0.30 | 0.53 | 0.06 | 3.84 | 0.32 | 136 | 2.87 | 99 |
| *Macroglossinae_Ampelophaga_rubiginosa* | 1.58 | 0.38 | 0.58 | 0.15 | 3.43 | 0.25 | 12 | 0.25 | 10 |
| *Macroglossinae_Angonyx_testacea* | 0.77 | 0.09 | 0.54 | 0.01 | 2.88 | 0.13 | 2 | 0.04 | 2 |
| *Macroglossinae_Cechenena_aegrota* | 1.22 | 0.26 | 0.56 | 0.09 | 3.16 | 0.16 | 33 | 0.70 | 30 |
| *Macroglossinae_Cechenena_helops* | 2.51 | 0.45 | 0.79 | 0.11 | 3.44 | 0.58 | 10 | 0.21 | 9 |
| *Macroglossinae_Cechetra_lineosa* | 2.07 | 0.44 | 0.57 | 0.08 | 3.72 | 0.24 | 806 | 17.04 | 523 |
| *Macroglossinae_Cechetra_minor* | 1.46 | 0.22 | 0.60 | 0.11 | 3.51 | 0.23 | 29 | 0.61 | 23 |
| *Macroglossinae_Cechetra_scotti* | 2.45 | 0.53 | 0.61 | 0.09 | 3.83 | 0.22 | 180 | 3.80 | 125 |
| *Macroglossinae_Cechetra_subangustata* | 2.23 | 0.46 | 0.57 | 0.08 | 3.77 | 0.29 | 222 | 4.69 | 179 |
| *Macroglossinae_Daphnis_hypothous* | 3.47 | 1.32 | 0.89 | 0.26 | 3.57 | 0.21 | 11 | 0.23 | 11 |
| *Macroglossinae_Eupanacra_busiris* | 0.76 | NA | 0.76 | NA | 5.75 | NA | 1 | 0.02 | 1 |
| *Macroglossinae_Eupanacra_perfecta* | 0.54 | 0.11 | 0.58 | 0.14 | 3.61 | 0.35 | 17 | 0.36 | 15 |
| *Macroglossinae_Eupanacra_sinuata* | 0.79 | 0.20 | 0.56 | 0.10 | 3.47 | 0.21 | 163 | 3.45 | 112 |
| *Macroglossinae_Eupanacra_variolosa* | 0.66 | NA | 0.70 | NA | 4.20 | NA | 1 | 0.02 | 1 |
| *Macroglossinae_Hippotion_boerhaviae* | 0.57 | 0.08 | 0.53 | 0.09 | 3.67 | 0.19 | 17 | 0.36 | 13 |
| *Macroglossinae_Hippotion_celerio* | 0.86 | 0.09 | 0.63 | 0.05 | 3.69 | 0.19 | 6 | 0.13 | 3 |
| *Macroglossinae_Macroglossum_species1* | NA | NA | NA | NA | NA | NA | 1 | 0.01 | 0 |
| *Macroglossinae_Macroglossum_species2* | 1.26 | 0.35 | 1.01 | 0.16 | 3.28 | 0.31 | 3 | 0.06 | 2 |
| *Macroglossinae_Nephele_hespera* | 1.29 | 0.36 | 0.71 | 0.21 | 3.09 | 0.15 | 18 | 0.38 | 17 |
| *Macroglossinae_Pergesa_acteus* | 1.21 | 0.23 | 0.75 | 0.16 | 3.51 | 0.21 | 13 | 0.27 | 12 |
| *Macroglossinae_Rhagastis_acuta* | 0.79 | 0.16 | 0.55 | 0.11 | 3.02 | 0.23 | 11 | 0.23 | 10 |
| *Macroglossinae_Rhagastis_albomarginatus* | 1.32 | 0.32 | 0.48 | 0.07 | 3.38 | 0.12 | 38 | 0.80 | 16 |
| *Macroglossinae_Rhagastis_castor* | 1.11 | 0.31 | 0.49 | 0.09 | 3.19 | 0.21 | 57 | 1.20 | 39 |
| *Macroglossinae_Rhagastis_confusa* | 1.06 | 0.25 | 0.45 | 0.07 | 3.32 | 0.27 | 102 | 2.16 | 64 |
| *Macroglossinae_Rhagastis_gloriosa* | 1.21 | 0.26 | 0.46 | 0.07 | 3.02 | 0.30 | 64 | 1.35 | 54 |
| *Macroglossinae_Rhagastis_lunata* | 1.51 | 0.35 | 0.56 | 0.10 | 3.07 | 0.17 | 111 | 2.35 | 88 |
| *Macroglossinae_Rhagastis_olivacea* | 1.01 | 0.18 | 0.48 | 0.07 | 3.17 | 0.28 | 66 | 1.40 | 39 |
| *Macroglossinae_Rhagastis_velata* | 0.95 | 0.25 | 0.59 | 0.12 | 2.95 | 0.14 | 5 | 0.11 | 3 |
| *Macroglossinae_Theretra_alecto* | 1.72 | 0.42 | 0.66 | 0.17 | 3.35 | 0.18 | 20 | 0.42 | 14 |
| *Macroglossinae_Theretra_boisduvalii* | 2.21 | 0.46 | 0.67 | 0.12 | 3.56 | 0.23 | 226 | 4.78 | 174 |
| *Macroglossinae_Theretra_clotho* | 2.01 | 0.43 | 0.79 | 0.18 | 3.60 | 0.17 | 179 | 3.78 | 140 |
| *Macroglossinae_Theretra_griseomarginata* | 0.50 | 0.05 | 0.44 | 0.03 | 2.88 | 0.22 | 4 | 0.08 | 2 |
| *Macroglossinae_Theretra_latreillii* | 1.15 | 0.25 | 0.58 | 0.12 | 3.23 | 0.18 | 5 | 0.11 | 5 |
| *Macroglossinae_Theretra_nessus* | 2.99 | 0.57 | 0.69 | 0.12 | 3.57 | 0.19 | 40 | 0.85 | 33 |
| *Macroglossinae_Theretra_oldenlandiae* | 0.79 | 0.16 | 0.50 | 0.06 | 3.54 | 0.17 | 19 | 0.40 | 15 |
| *Macroglossinae_Theretra_pallicosta* | 1.49 | 0.35 | 0.66 | 0.14 | 3.43 | 0.16 | 8 | 0.17 | 5 |
| *Macroglossinae_Theretra_suffusa* | 1.45 | NA | 0.70 | NA | 3.62 | NA | 1 | 0.02 | 1 |
| *Smerinthinae_Ambulyx_liturata* | 2.47 | 0.51 | 0.48 | 0.07 | 3.93 | 0.40 | 123 | 2.60 | 64 |
| *Smerinthinae_Ambulyx_maculifera* | 1.66 | 0.41 | 0.46 | 0.11 | 3.60 | 0.17 | 20 | 0.42 | 12 |
| *Smerinthinae_Ambulyx_moorei* | 1.48 | 0.24 | 0.41 | 0.02 | 3.57 | 0.13 | 2 | 0.04 | 2 |
| *Smerinthinae_Ambulyx_ochracea* | 1.13 | 0.26 | 0.38 | 0.07 | 3.62 | 0.24 | 101 | 2.13 | 73 |
| *Smerinthinae_Ambulyx_pseudoclavata* | 1.92 | 0.52 | 0.42 | 0.11 | 3.52 | 0.27 | 10 | 0.21 | 9 |
| *Smerinthinae_Ambulyx_sericeipennis* | 2.22 | 0.15 | 0.39 | 0.02 | 3.59 | 0.20 | 5 | 0.11 | 3 |
| *Smerinthinae_Ambulyx_substrigilis* | 1.73 | 0.34 | 0.43 | 0.05 | 3.43 | 0.19 | 48 | 1.01 | 28 |
| *Smerinthinae_Ambulyx_tobii* | 2.24 | 0.36 | 0.46 | 0.04 | 3.55 | 0.16 | 34 | 0.72 | 21 |
| *Smerinthinae_Amplypterus_mansoni* | 4.19 | 0.53 | 0.63 | 0.07 | 3.58 | 0.18 | 33 | 0.70 | 13 |
| *Smerinthinae_Amplypterus_panopus* | 3.22 | 0.71 | 0.53 | 0.01 | 3.48 | 0.01 | 3 | 0.06 | 2 |
| *Smerinthinae_Callambulyx_junonia* | 1.56 | 0.29 | 0.44 | 0.05 | 3.26 | 0.20 | 7 | 0.15 | 4 |
| *Smerinthinae_Callambulyx_poecilus* | 0.88 | 0.22 | 0.45 | 0.09 | 3.71 | 0.32 | 14 | 0.30 | 10 |
| *Smerinthinae_Callambulyx_rubricosa* | 2.75 | 0.77 | 0.56 | 0.13 | 3.64 | 0.42 | 46 | 0.97 | 26 |
| *Smerinthinae_Clanis_schwartzi* | NA | NA | NA | NA | NA | NA | 1 | 0.01 | 0 |
| *Smerinthinae_Clanis_titan* | NA | NA | NA | NA | NA | NA | 1 | 0.01 | 0 |
| *Smerinthinae_Clanis_undulosa* | 4.87 | 0.93 | 0.81 | 0.17 | 5.06 | 0.79 | 30 | 0.63 | 13 |
| *Smerinthinae_Craspedortha_porphyria* | 0.51 | 0.13 | 0.63 | 0.32 | 3.25 | 1.09 | 21 | 0.44 | 15 |
| *Smerinthinae_Cypa_decolor* | 0.75 | NA | 0.68 | NA | 3.25 | NA | 1 | 0.02 | 1 |
| *Smerinthinae_Daphnusa_sinocontinentalis* | 1.13 | 0.24 | 0.43 | 0.10 | 2.79 | 0.17 | 7 | 0.15 | 6 |
| *Smerinthinae_Dolbina_inexacta* | 1.28 | 0.19 | 0.58 | 0.07 | 3.36 | 0.20 | 14 | 0.30 | 11 |
| *Smerinthinae_Marumba_cristata* | 2.56 | 0.72 | 0.51 | 0.11 | 3.35 | 0.34 | 15 | 0.32 | 9 |
| *Smerinthinae_Marumba_dyras* | 2.19 | 0.46 | 0.56 | 0.11 | 3.23 | 0.31 | 34 | 0.72 | 30 |
| *Smerinthinae_Marumba_echephron* | 2.32 | 0.81 | 0.62 | 0.19 | 3.85 | 0.81 | 11 | 0.23 | 4 |
| *Smerinthinae_Marumba_spectabilis* | 2.10 | 0.08 | 0.73 | 0.15 | 3.48 | 0.99 | 2 | 0.04 | 2 |
| *Smerinthinae_Rhodoprasina_species* | 1.50 | 0.23 | 0.53 | 0.07 | 3.81 | 0.28 | 9 | 0.19 | 5 |
| *Sphinginae_Acherontia_lachesis* | 4.47 | 1.16 | 1.12 | 0.22 | 3.98 | 0.62 | 41 | 0.87 | 26 |
| *Sphinginae_Acherontia_styx* | 2.49 | 0.19 | 0.84 | 0.38 | 3.95 | 0.73 | 3 | 0.06 | 2 |
| *Sphinginae_Agrius_convolvuli* | 2.06 | 0.63 | 0.88 | 0.25 | 4.36 | 0.89 | 60 | 1.27 | 14 |
| *Sphinginae_Apocalypsis_velox* | 3.76 | 0.99 | 0.63 | 0.14 | 3.58 | 0.21 | 35 | 0.74 | 29 |
| *Sphinginae_Megacorma_obliqua* | 4.25 | 0.30 | 0.92 | 0.05 | 3.35 | 0.31 | 3 | 0.06 | 3 |
| *Sphinginae_Meganoton_analis* | 4.08 | 0.71 | 0.57 | 0.09 | 3.44 | 0.22 | 66 | 1.40 | 47 |
| *Sphinginae_Meganoton_rubescens* | 2.75 | 0.77 | 0.50 | 0.09 | 3.20 | 0.21 | 28 | 0.59 | 20 |
| *Sphinginae_Psilogramma_increta* | 3.39 | 0.55 | 0.64 | 0.09 | 3.63 | 0.11 | 7 | 0.15 | 6 |
| *Sphinginae_Psilogramma_menephron* | 3.16 | 0.88 | 0.63 | 0.09 | 3.49 | 0.44 | 55 | 1.16 | 41 |

**
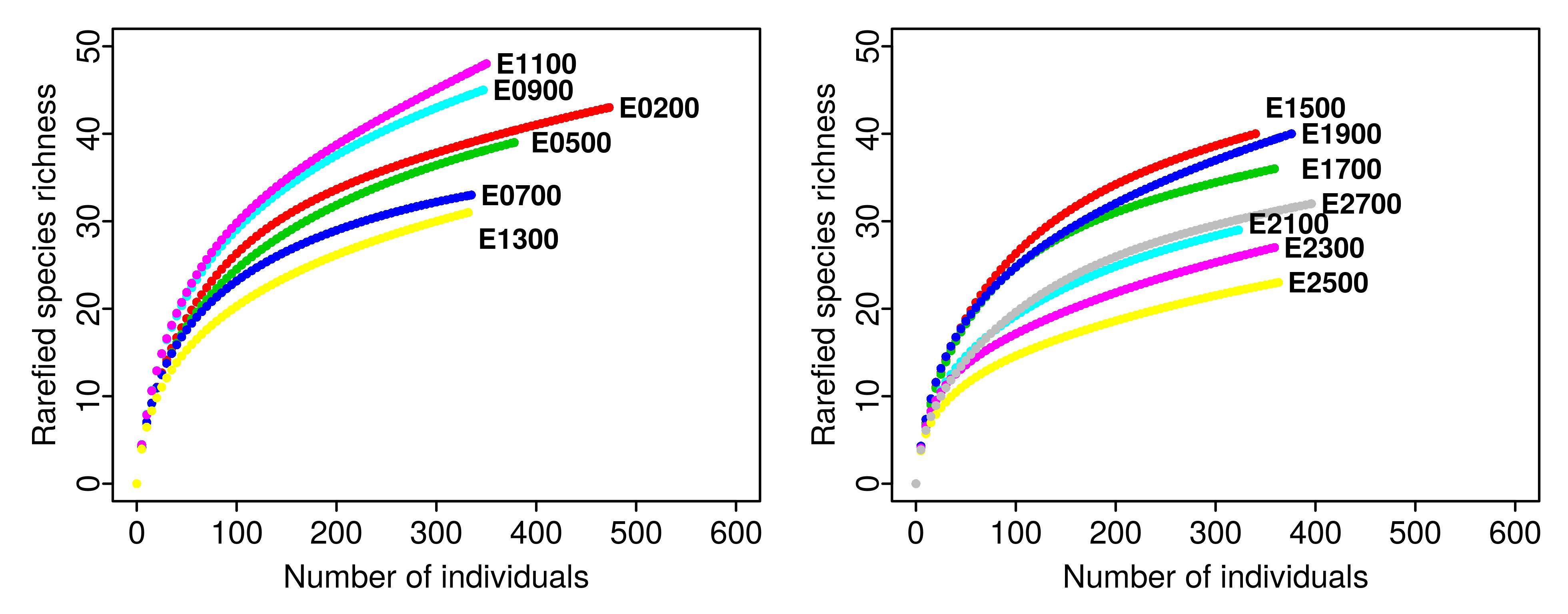
**

**Figure A2:** **Hawkmoth taxonomic diversity rarefaction curves.**

**B: Hawkmoth Traits**

We obtained shape and size calibrated images by photographing moths against a reference rectangular grid on the UV light screen (Figure S1.2). The distortion of this background rectangular grid (of known dimensions) was used to calibrate the image for shape and size. We measured the following traits on the calibrated image using the 8 landmarks A-H in Figure S1.2:

1. Body: Body length = LAB, Thorax width = LCF
2. Right wing: costum = LCD , termen = LDE, dorsum = LCE
3. Left wing: costum = LFG, termen = LGH, dorsum = LFH

We modeled body volume as a spindle shaped bicone and multiplied the same by the density of water (1 g cm^–3^) to obtain body mass.

$BodyVolume\left( {mm}^{3} \right)=\frac{1}{3}*\Pi\left( \frac{ThoraxWidth}{2} \right)^{2}\left( {mm}^{2} \right)*BodyLength\left( mm \right)$,

Wing area was calculated from the triangle defined by the 3 landmarks CDE and FGH.

$WingArea\left( {mm}^{2} \right)=\sqrt{\left( \left( s*\left( s-L_{costum} \right)*\left( s-L_{termen} \right)*\left( s-L_{dorsum} \right) \right) \right)}$

$s=\frac{L_{costum}+L_{termen}+L_{dorsum}}{2}$where,

$WingLoad\left( \frac{gm}{{mm}^{2}} \right)=\frac{BodyMass\left( gm \right)}{WingArea\left( {mm}^{2} \right)}$

Wing-loading was obtained from:

.

$AspectRatio=\frac{L_{costum}^{2}\left( mm \right)}{2WingArea\left( mm \right)}$

Wing aspect-ratio was obtained from:

.

The discrepancy between left and right wing dimensions were used to identify poorly calibrated images. They were subsequently averaged for all analysis. The details of the method for image calibration is described in Mungee & Athreya (2019)


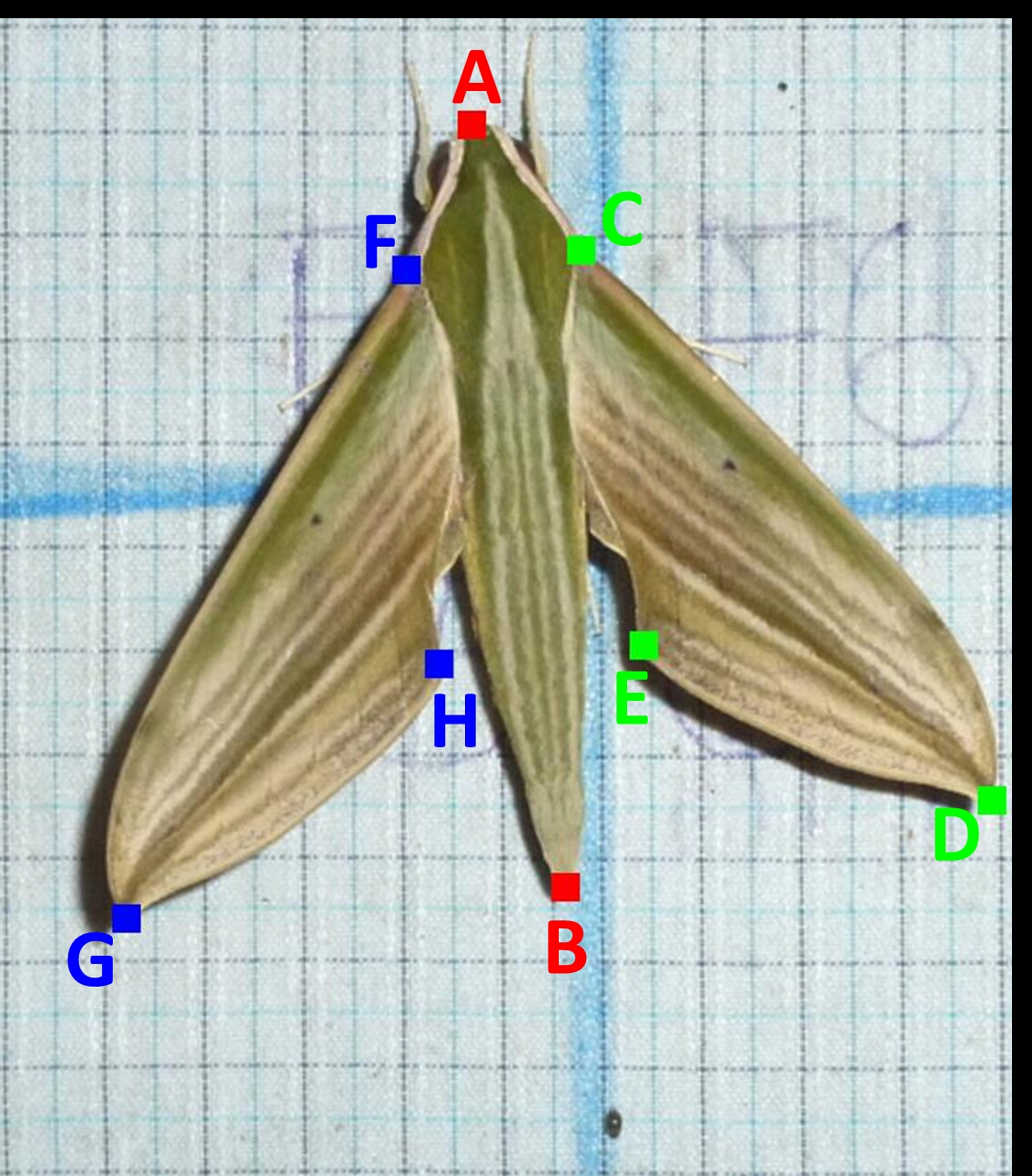


**Figure B1:** **Trait data images of hawkmoths**


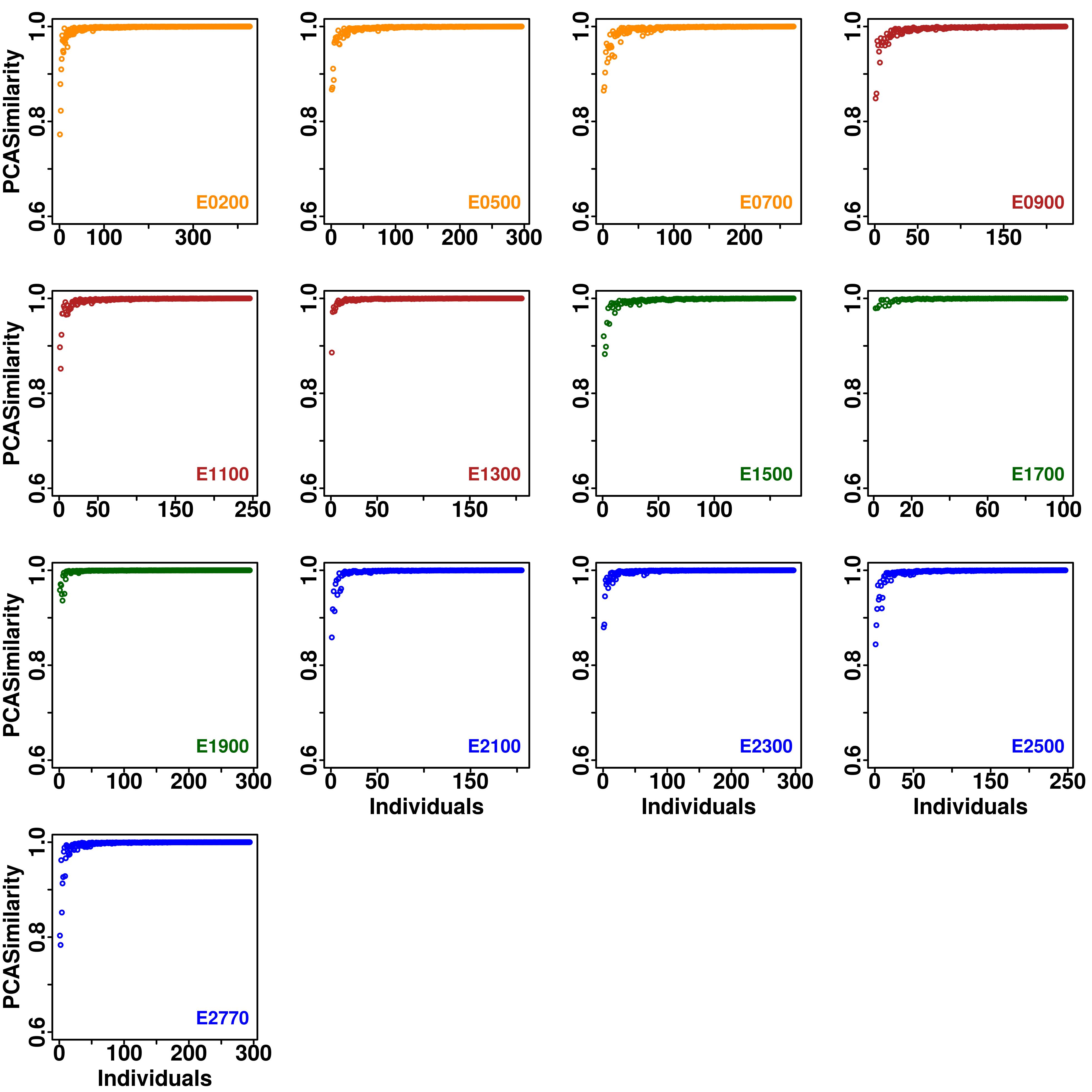
Individuals were imaged against a reference background grid used as UV illuminated light screen. The rectangular grids were used to calibrate the image for shape and size. The 8 landmarks A-H were used to measure the body and wing dimensions.


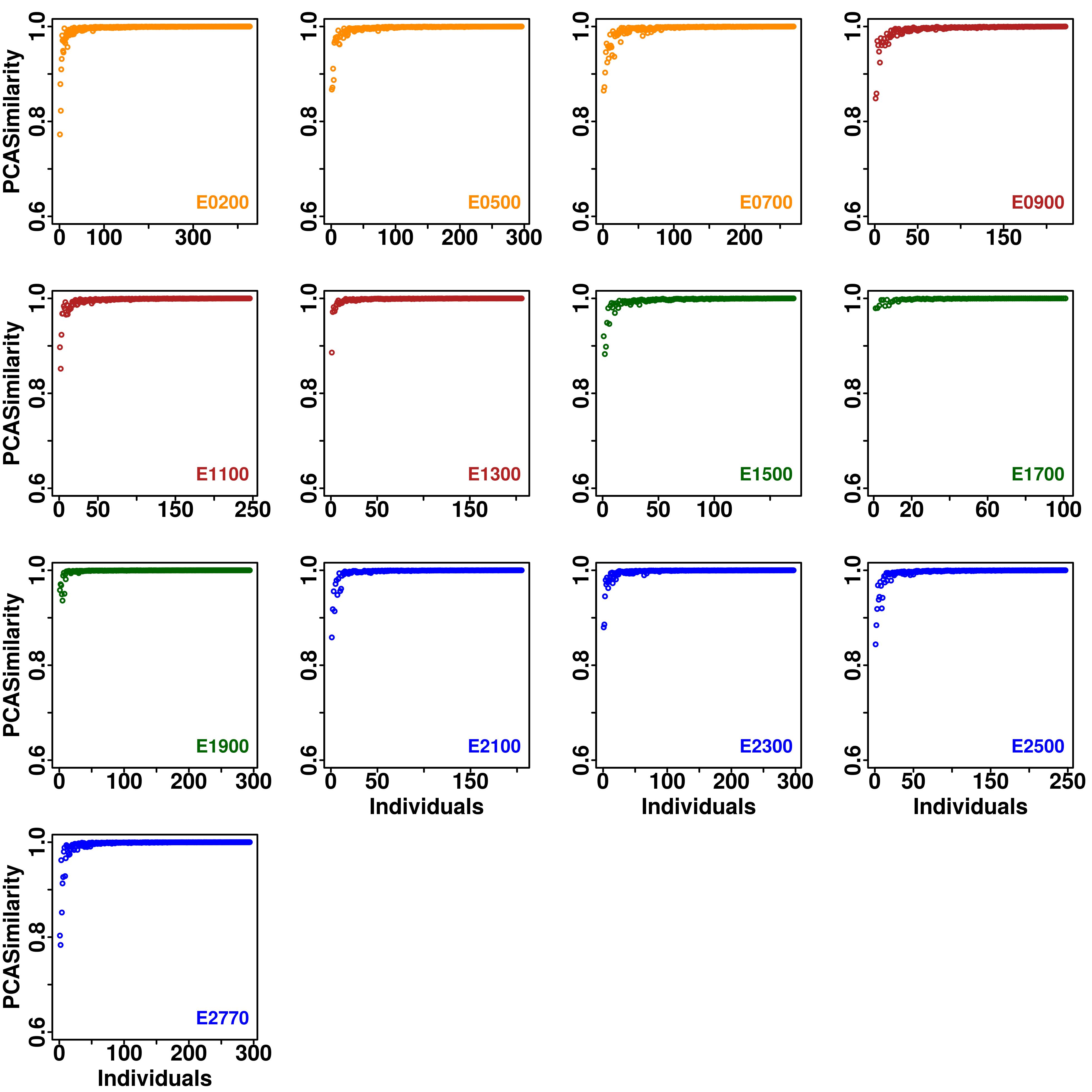


**Figure B2: Hawkmoth functional diversity rarefaction curves.**

Each plot represents a different elevational community. It can be seen that the overall trait volume is quickly achieved by sample size 50-100. The rest of the data essentially fill in the “intraspecific variability” space. The traits were normalized using community-specific mean and standard deviations prior to analysis. The rarefactions were performed using function *Rarefaction* from package *evolqg* (Melo et al. 2015, R core development Team, 2013).

**C: Environmental gradient**

We used 4 environmental variables including mean annual temperature (MAT), mean annual precipitation (APPT), productivity (EVI: enhanced vegetation index, and NDVI: normalised vegetation index) and air density (AD). MAT and APPT with a spatial resolution of 1 km^2^ were downloaded from ***worldclim*** (https://www.worldclim.org/) for the years 2004-2014. EVI and NDVI were obtained from NASA’s MODIS satellite products (MOD13Q1) with a resolution of 250 m. Poor pixel quality values were removed before arriving at elevation specific mean values. Air density was calculated for the elevational communities using a global pressure profile and local temperature values using:

$AirDensity=\left[ P_{std}*1-\left( \frac{L*E}{T_{std}} \right)^{\left( \frac{gM}{R*T} \right)} \right]*\frac{M}{R*T}$

where,

P_Std_ = sea level standard atmospheric pressure, 101325 [Pa](https://en.wikipedia.org/wiki/Pascal_(unit))

L = temperature lapse rate, 0.0065 K/m

E = elevation (in meters above sea level) for the sampling location

T_Std_ = standard temperature at sea level, 288.15 K

g = earth-surface gravitational acceleration, 9.80665 m/s²

M = molar mass of dry air, 0.0289644 kg/mol

R = universal gas constant, 8.31447 J/(mol.K)

T = temperature for the sampling location (K; Obtained from worldclim)

EVI was preferred to NDVI since the two are strongly correlated and EVI has higher sensitivity at higher biomass (r^2^ = 0.62, p < 0.001)

Temperature, precipitation and air density were very strongly correlated with elevation (R^2^ = 0.95-0.98). This is not surprising since the interpolation used to construct the fine scale temperature and precipitation WorldClim surfaces is likely to be dominated by the elevation variable within a compact and sparsely sampled region (50 km^2^) like our study site. Air density was calculated from temperature and elevation (above formula). This is also seen in the PCA plot (Suppl. Figure S1.4) in which the three variables are essentially in the same direction while productivity (EVI) is perpendicular. Effectively, the composite variable *Environment* is dominated by elevation (Suppl. Figure S1.5), with some contribution from productivity. Productivity (EVI) was also correlated with elevation but to a lesser degree (R2 = 0.53). Figure S1.3 shows the different relationships.

We used a principal component analysis to investigate the possibility of constructing a composite environmental variable (Tavble S1.4; Figure S1.4). The first two principal components explained 91.4 % (PC1) and 7.7 % (PC2) of the variance. Figure S1.4 shows that PC1 consists of temperature, precipitation and air density vectors, all pointing essentially in the same direction on account of their link to elevation, along with some contribution from productivity which is normal to the other three. PC1 was found to be highly correlated with elevation (R2 = 0.95; Figure S1.5).


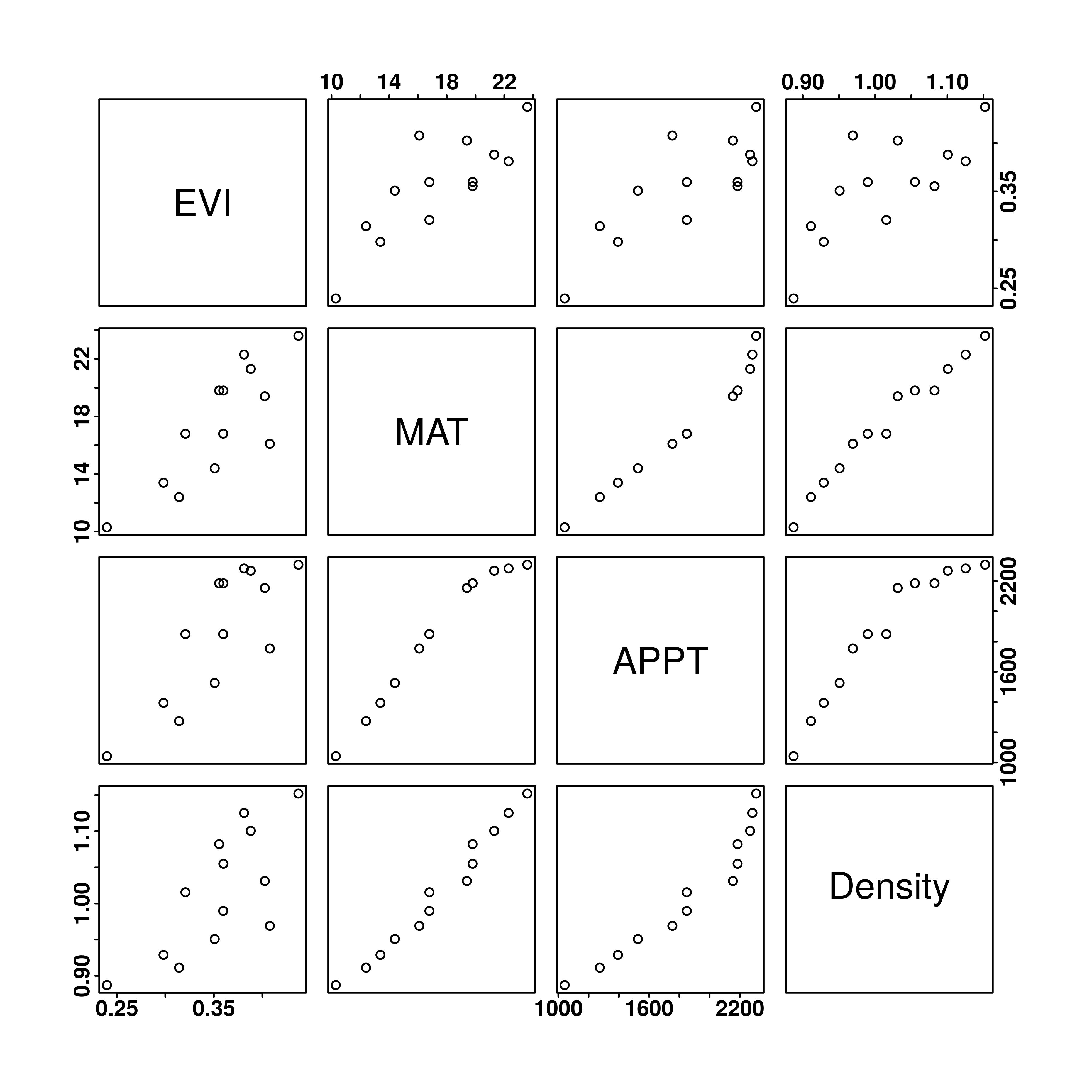


| **Model** | **Slope** | **Intercept** | **R.squared** | **p.value** |
| --- | --- | --- | --- | --- |
| EVI ~ Elevation | -5.04 x 10^-5^ | 4.31 x 10^-1^ | 0.53 | < 0.005 |
| MAT ~ Elevation | -5.10 x 10^-3^ | 2.51 x 10^1^ | 0.98 | < 0.005 |
| APPT ~ Elevation | -5.25 x 10^-1^ | 2.64 x 10^3^ | 0.92 | < 0.005 |
| AD ~ Elevation | -1.1 x 10^-4^ | 0.12 x 10^1^ | 0.99 | < 0.005 |
| EVI ~ APPT | 0.66 x 10^4^ | -4.91 x 10^2^ | 0.62 | < 0.005 |
| EVI ~ AD | 4.6 x 10^-1^ | -1.12 x 10^-1^ | 0.50 | < 0.005 |

**Figure C1: Relationships between the environmental variables.**

The environmental variables are EVI: enhanced vegetation index, MAT: mean annual temperature, APPT, mean annual precipitation, and air density. Linear regression coefficients between the variables are listed in the table above.

**Table C1: Environmental variables of sampled communities**

The columns are Plot: elevational community, MAT: mean annual temperature, APPT: mean annual precipitation, EVI: enhanced vegetation index (productivity), AD: air density, and their scores on PC1: first principle component, and PC2: second principal component

| **Plot** | **MAT** | **APPT** | **EVI** | **AD** | **Score on PC1** | **Score on PC2** |
| --- | --- | --- | --- | --- | --- | --- |
| **E0200** | 23.6 | 2308 | 0.4373 | 1.152 | -2.88 | -0.21 |
| **E0500** | 22.3 | 2283 | 0.3812 | 1.125 | -2.03 | 0.52 |
| **E0700** | 21.3 | 2268 | 0.3881 | 1.101 | -1.80 | 0.23 |
| **E0900** | 19.8 | 2185 | 0.3555 | 1.082 | -1.11 | 0.57 |
| **E1100** | 19.8 | 2185 | 0.3599 | 1.055 | -0.99 | 0.36 |
| **E1300** | 19.4 | 2154 | 0.4026 | 1.031 | -1.12 | -0.50 |
| **E1500** | 16.8 | 1849 | 0.3207 | 1.016 | 0.38 | 0.54 |
| **E1700** | 16.8 | 1849 | 0.3597 | 0.990 | 0.20 | -0.24 |
| **E1900** | 16.1 | 1754 | 0.4078 | 0.969 | 0.11 | -1.21 |
| **E2100** | 14.4 | 1526 | 0.3509 | 0.951 | 1.21 | -0.53 |
| **E2300** | 13.4 | 1394 | 0.2981 | 0.929 | 2.10 | 0.14 |
| **E2500** | 12.4 | 1274 | 0.3142 | 0.911 | 2.33 | -0.31 |
| **E2770** | 10.3 | 1042 | 0.2397 | 0.895 | 3.63 | 0.65 |


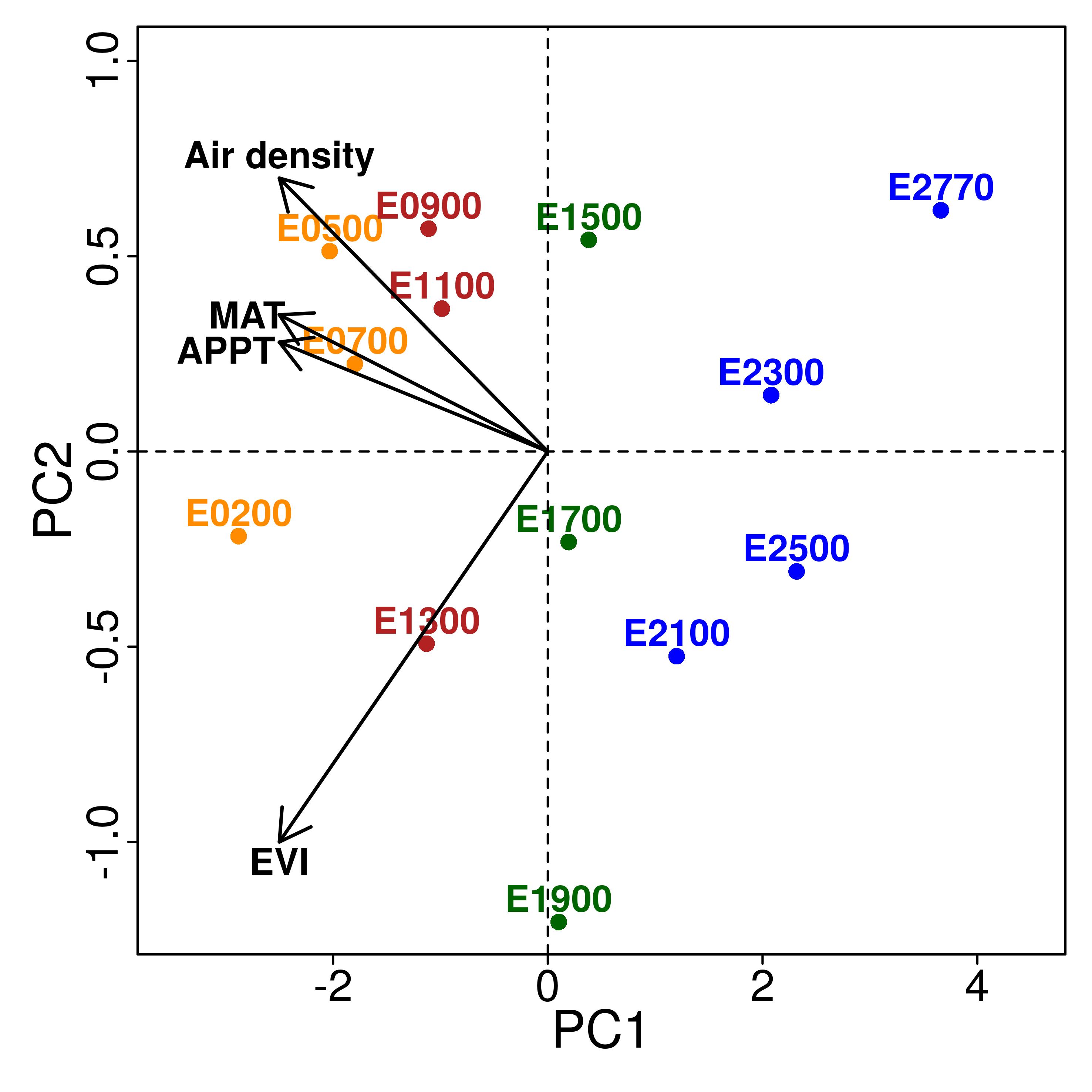


**Figure C2: Principal component analysis of environmental variables**

The environmental variables are: AD Air density, APPT mean annual precipitation, EVI Enhanced vegetation index (productivity), and MAT Mean Annual Temperature. The percentage contribution of each component to PC1 is listed in paranthesis.

|  | **PC1** | **PC2** | **PC3** | **PC4** |
| --- | --- | --- | --- | --- |
| **EVI** (22.96%) | -0.459 | -0.869 | -0.146 | -0.113 |
| **MAT** (26.01%) | -0.520 | 0.186 | -0.115 | 0.825 |
| **APPT** (25.71%) | -0.514 | 0.169 | 0.803 | -0.250 |
| **AD** (25.33%) | -0.505 | 0.425 | -0.566 | -0.493 |


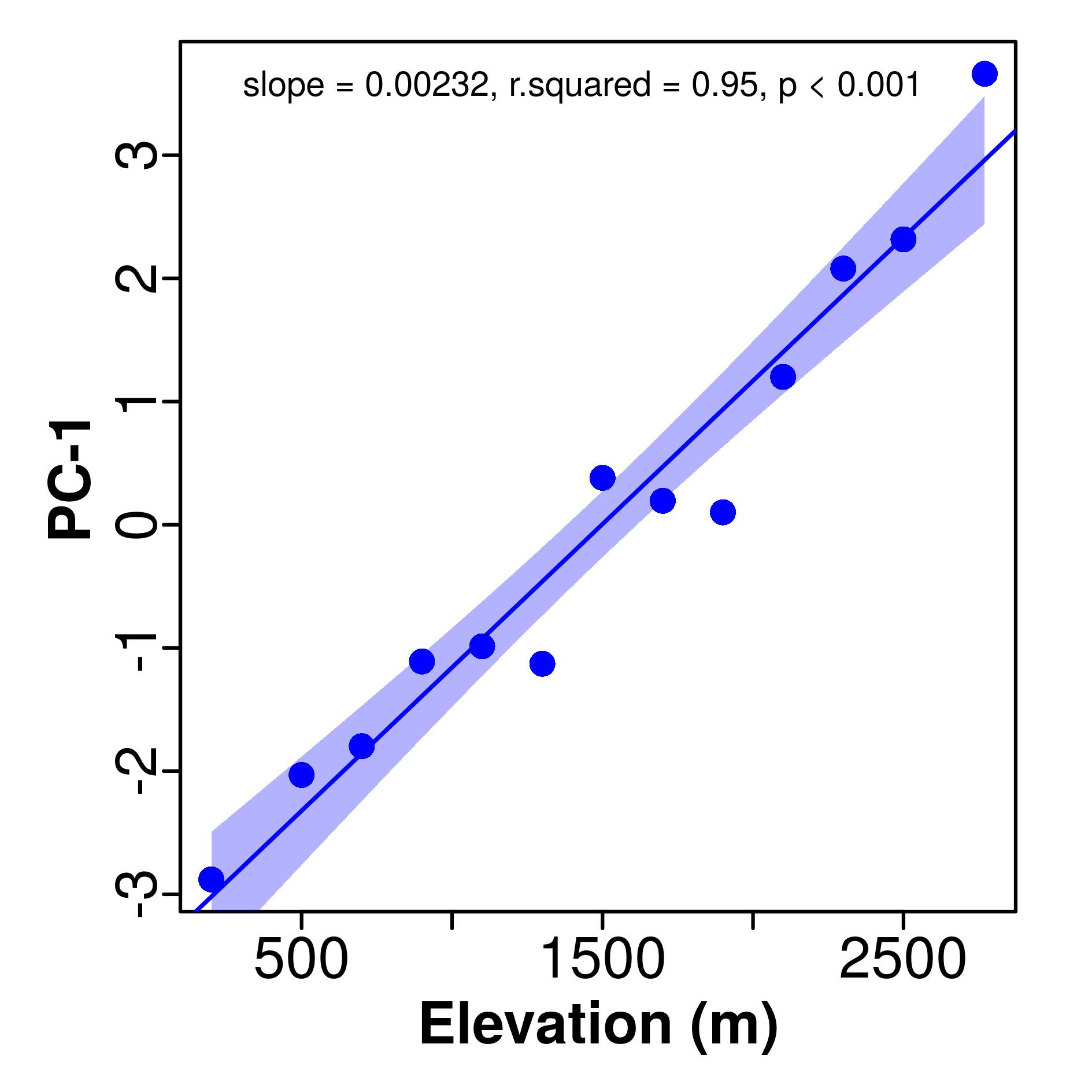


**Figure C3: Relationship between PC1 and elevation**

**D: *T-statsitics***

**Table D1:** Null models used to calculate the statistical significance for *T-statistics* (adapted from Taudière & Violle 2015).

|  | **Null model** | **Null hypothesis** | **Randomization** | **Alternate hypothesis** |
| --- | --- | --- | --- | --- |
| *T_IP/IC_* | local | Trait value distribution is independent of species identity  there is no internal filtering. | Individual trait values are shuffled within each community | two individuals belonging to a population have more similar trait values than two individuals drawn randomly from the community |
| *T_I/CIR_* | Regional. ind | Individual trait value  distribution is drawn randomly from the regional pool:  there is no external filtering acting on individuals. | Individual trait values are shuffled within the regional pool, keeping the number of individuals in each community constant. | Two individuals belonging to a community have more similar trait values than two individuals drawn randomly from the regional pool |
| *T_PC/PR_* | Regional. pop | Species mean trait value distribution is drawn randomly from the regional pool  there is no external filtering acting on species. | Each individual is assigned the mean value of the species; the values are then shuffled within the regional pool, keeping the number of individuals in each community constant. | Two individuals belonging to a community have more similar population based trait values than two individuals drawn randomly from the regional pool while taking abundance into account |

**Table D2:** **Observed *T-statistics* for elevational communities of hawkmoths.**

The columns are elevation of the community, average variance within populations in a community (σ2IP), variance within the community (σ2IC), internal filter metric (TIP/IC), external filter metric using individual traits (TIC/IR) and external filter using population means (TPC/PR). The traits used are body mass (BM), wing loading (WL) and wing aspect ratio (AR). The variance values listed in the table are to be multiplied by 10^2^ to get the actual values.

| Elev- ation | σ2IP (10^–2^) | | | σ2IC (10^–2^) | | | TIP/IC | | | TIC/IR | | | TPC/PR | | |
| --- | --- | --- | --- | --- | --- | --- | --- | --- | --- | --- | --- | --- | --- | --- | --- |
|  | **BM** | **WL** | **AR** | **BM** | **WL** | **AR** | **BM** | **WL** | **AR** | **BM** | **WL** | **AR** | **BM** | **WL** | **AR** |
| **200** | 0.809 | 0.683 | 0.098 | 3.131 | 1.701 | 0.129 | 0.26 | 0.40 | 0.76 | 0.84 | 1.71 | 0.62 | 0.73 | 1.14 | 0.49 |
| **500** | 0.662 | 0.371 | 0.167 | 2.647 | 0.884 | 0.220 | 0.25 | 0.42 | 0.76 | 0.71 | 0.89 | 1.05 | 0.82 | 1.10 | 0.92 |
| **700** | 0.811 | 0.688 | 0.164 | 3.502 | 1.062 | 0.307 | 0.23 | 0.65 | 0.53 | 0.95 | 1.07 | 1.47 | 0.77 | 0.87 | 1.34 |
| **900** | 0.908 | 0.448 | 0.097 | 2.724 | 1.239 | 0.180 | 0.33 | 0.36 | 0.54 | 0.74 | 1.24 | 0.86 | 0.76 | 0.99 | 0.54 |
| **1100** | 0.95 | 0.606 | 0.154 | 4.266 | 0.875 | 0.220 | 0.22 | 0.69 | 0.70 | 1.15 | 0.88 | 1.05 | 1.28 | 1.03 | 1.67 |
| **1300** | 0.765 | 0.333 | 0.109 | 4.357 | 0.542 | 0.231 | 0.18 | 0.61 | 0.47 | 1.18 | 0.54 | 1.11 | 1.13 | 0.40 | 1.76 |
| **1500** | 0.991 | 0.399 | 0.077 | 3.439 | 0.834 | 0.159 | 0.29 | 0.48 | 0.48 | 0.93 | 0.84 | 0.76 | 1.18 | 1.15 | 0.60 |
| **1700** | 0.562 | 0.345 | 0.080 | 5.634 | 0.946 | 0.266 | 0.10 | 0.36 | 0.30 | 1.52 | 0.95 | 1.27 | 1.43 | 1.09 | 1.65 |
| **1900** | 0.469 | 0.398 | 0.080 | 4.708 | 0.686 | 0.212 | 0.10 | 0.58 | 0.38 | 1.27 | 0.69 | 1.02 | 1.08 | 0.98 | 1.09 |
| **2100** | 0.827 | 0.533 | 0.110 | 4.226 | 0.650 | 0.210 | 0.20 | 0.82 | 0.53 | 1.14 | 0.65 | 1.00 | 1.12 | 0.77 | 0.61 |
| **2300** | 0.927 | 0.338 | 0.083 | 3.631 | 0.592 | 0.205 | 0.26 | 0.57 | 0.40 | 0.98 | 0.59 | 0.98 | 1.13 | 0.77 | 0.86 |
| **2500** | 0.648 | 0.406 | 0.089 | 2.586 | 0.688 | 0.163 | 0.25 | 0.59 | 0.55 | 0.70 | 0.69 | 0.78 | 0.88 | 0.71 | 0.59 |
| **2770** | 0.596 | 0.485 | 0.129 | 2.853 | 0.705 | 0.212 | 0.21 | 0.70 | 0.62 | 0.77 | 0.71 | 1.01 | 0.89 | 1.10 | 1.08 |

**Table D3:** **Standard Effect Size for the three *T-statsitic* metrics for elevational communities of hawkmoths.**

The columns are elevation of the community, observed SES, 2.75 percentile of the null distribution (low), 97.5 percentile of the null distribution (high). The calculations were made separately for body mass (BM), wing loading (WL) and wing aspect ratio (AR).

| **Elev** | **BM** | **BM_low** | **BM_high** | **WL** | **WL_low** | **WL_high** | **AR** | **AR_low** | **AR_high** |
| --- | --- | --- | --- | --- | --- | --- | --- | --- | --- |
| **T_IP/IC_** |  |  |  |  |  |  |  |  |  |
| **200** | -5.67 | -1.63 | 2.24 | -4.64 | -1.68 | 2.19 | -1.48 | -1.59 | 2.27 |
| **500** | -4.64 | -1.59 | 2.25 | -3.36 | -1.61 | 2.17 | -1.38 | -1.54 | 2.21 |
| **700** | -5.27 | -1.52 | 2.27 | -2.13 | -1.45 | 2.35 | -2.75 | -1.60 | 2.41 |
| **900** | -4.13 | -1.70 | 2.10 | -3.81 | -1.68 | 2.14 | -2.37 | -1.49 | 2.30 |
| **1100** | -5.62 | -1.66 | 2.31 | -2.20 | -1.67 | 2.17 | -1.81 | -1.58 | 2.24 |
| **1300** | -5.47 | -1.71 | 2.34 | -2.45 | -1.63 | 2.46 | -3.06 | -1.55 | 2.41 |
| **1500** | -3.87 | -1.67 | 2.11 | -2.85 | -1.63 | 2.20 | -2.42 | -1.56 | 2.41 |
| **1700** | -4.27 | -1.61 | 2.23 | -2.99 | -1.67 | 2.20 | -2.92 | -1.59 | 2.39 |
| **1900** | -6.13 | -1.64 | 2.36 | -2.71 | -1.60 | 2.28 | -3.58 | -1.47 | 2.26 |
| **2100** | -4.59 | -1.66 | 2.31 | -1.03 | -1.55 | 2.30 | -2.28 | -1.41 | 2.54 |
| **2300** | -3.87 | -1.40 | 2.21 | -2.39 | -1.55 | 2.59 | -2.94 | -1.38 | 2.51 |
| **2500** | -3.54 | -1.43 | 2.47 | -1.91 | -1.43 | 2.50 | -1.80 | -1.27 | 2.67 |
| **2770** | -3.75 | -1.52 | 2.34 | -1.43 | -1.49 | 2.38 | -1.61 | -1.54 | 2.37 |
| **T_IC/IR_** |  |  |  |  |  |  |  |  |  |
| **200** | -2.18 | -1.91 | 1.97 | 8.93 | -1.94 | 1.94 | -3.97 | -1.73 | 2.04 |
| **500** | -3.17 | -1.95 | 2.04 | -1.18 | -1.82 | 2.08 | 0.49 | -1.72 | 1.99 |
| **700** | -0.60 | -1.84 | 2.08 | 0.57 | -1.90 | 2.07 | 3.67 | -1.86 | 2.05 |
| **900** | -2.57 | -1.94 | 1.98 | 2.24 | -1.87 | 2.04 | -0.94 | -1.75 | 2.17 |
| **1100** | 1.51 | -1.80 | 2.08 | -1.07 | -1.84 | 2.01 | 0.38 | -1.74 | 2.04 |
| **1300** | 1.60 | -1.97 | 2.05 | -3.92 | -1.75 | 2.20 | 0.69 | -1.74 | 2.27 |
| **1500** | -0.62 | -1.93 | 1.92 | -1.21 | -1.79 | 2.01 | -1.46 | -1.66 | 2.22 |
| **1700** | 3.52 | -1.89 | 2.07 | -0.30 | -1.80 | 2.03 | 1.33 | -1.64 | 2.26 |
| **1900** | 2.95 | -1.90 | 2.03 | -3.12 | -1.82 | 2.19 | 0.06 | -1.79 | 2.18 |
| **2100** | 1.31 | -1.99 | 1.90 | -2.98 | -1.82 | 2.23 | 0.01 | -1.76 | 2.06 |
| **2300** | -0.18 | -1.84 | 2.02 | -4.15 | -1.83 | 1.98 | -0.14 | -1.77 | 2.08 |
| **2500** | -3.09 | -1.89 | 2.11 | -2.78 | -1.86 | 1.99 | -1.66 | -1.74 | 2.27 |
| **2770** | -2.60 | -1.83 | 2.10 | -2.97 | -1.82 | 2.05 | 0.14 | -1.78 | 2.12 |
| **T_PC/PR_** |  |  |  |  |  |  |  |  |  |
| **200** | -0.60 | -1.57 | 2.28 | 0.69 | -1.44 | 2.33 | -1.19 | -1.34 | 2.86 |
| **500** | -0.43 | -1.57 | 2.39 | 0.41 | -1.40 | 2.44 | -0.06 | -1.40 | 2.65 |
| **700** | -0.06 | -1.44 | 2.40 | 0.18 | -1.22 | 2.73 | 1.49 | -1.18 | 2.51 |
| **900** | -0.60 | -1.51 | 2.34 | 0.07 | -1.39 | 2.46 | -1.08 | -1.33 | 2.68 |
| **1100** | 0.79 | -1.60 | 2.09 | 0.04 | -1.45 | 2.31 | 1.95 | -1.42 | 2.53 |
| **1300** | 0.29 | -1.39 | 2.25 | -1.18 | -1.33 | 2.25 | 1.59 | -1.21 | 2.59 |
| **1500** | 0.13 | -1.61 | 2.17 | -0.01 | -1.49 | 2.27 | -1.23 | -1.33 | 2.68 |
| **1700** | 0.51 | -1.51 | 2.30 | -0.16 | -1.30 | 2.40 | 0.86 | -1.23 | 2.44 |
| **1900** | 0.17 | -1.58 | 2.26 | -0.02 | -1.36 | 2.30 | 0.19 | -1.29 | 2.76 |
| **2100** | 0.40 | -1.53 | 2.31 | -0.41 | -1.39 | 2.38 | -0.79 | -1.24 | 2.70 |
| **2300** | 0.29 | -1.56 | 2.29 | -0.48 | -1.39 | 2.45 | -0.32 | -1.21 | 2.76 |
| **2500** | -0.30 | -1.33 | 2.38 | -0.59 | -1.26 | 2.70 | -0.84 | -1.14 | 3.00 |
| **2770** | -0.52 | -1.53 | 2.33 | 0.04 | -1.59 | 2.25 | 0.02 | -1.29 | 2.78 |

**E: Analyses with Diversity data set**

We repeated the three major analyses with modified data sets to understand the impact of incomplete data.

We could measure traits for only 3301 individuals (the trait data set) out of the total diversity data set of 4731 individuals. Therefore, we redid the analysis by filling in the missing trait data by randomly resampling the traits from others of the same species in the same community. For example, we could measure traits for only 66 of the 79 individuals of Acosmerycoides harterti at 700 m. The remaining 13 individuals were assigned trait values drawn randomly from the set of 66 individuals.

1. Table E1: to be compared with Main Table 1
2. Figure E1: to be compared with Main Figure 2
3. Figure E2: to be compared with Main, Figure 3
4. Figure E3: to be compared with Main, Figure 4

**Table E1: Linear regression of hawkmoth community traits with elevation**

(a) The community mean trait value was calculated using the population mean trait values weighted by local abundance.

(b) The overlap was measured for the trait kernel distributions of pairs of communities and regressed against the elevational separation between them.

|  | **Intercept ± SE** | **Slope ± SE** | **Adj. R^2^** | **p** |
| --- | --- | --- | --- | --- |
| **Community mean trait value**  **with elevation** | | | | |
| **Body mass** | 2.01 ± 0.04 | 0.06 ± 0.02 | 0.34 | **< 0.05** |
| Wing loading | (5.89 ± 0.11) x 10^-3^ | (-7.02 ± 6.01) x 10^-5^ | 0.03 | 0.27 |
| **Wing aspect ratio** | 3.53 ± 0.02 | 0.04 ± 0.01 | 0.51 | **< 0.005** |
| **Trait distribution overlap**  **with elevational separation** | | | | |
| **Body mass** | 0.87 ± 0.02 | (-6.97 ± 1.52) x 10^-5^ | 0.21 | **< 0.005** |
| **Wing loading** | 0.89 ± 0.02 | (-4.67 ± 1.53) x 10^-5^ | 0.10 | **< 0.005** |
| **Wing aspect ratio** | 0.88 ± 0.01 | (-4.78 ± 0.10) x 10^-5^ | 0.22 | **< 0.005** |

**
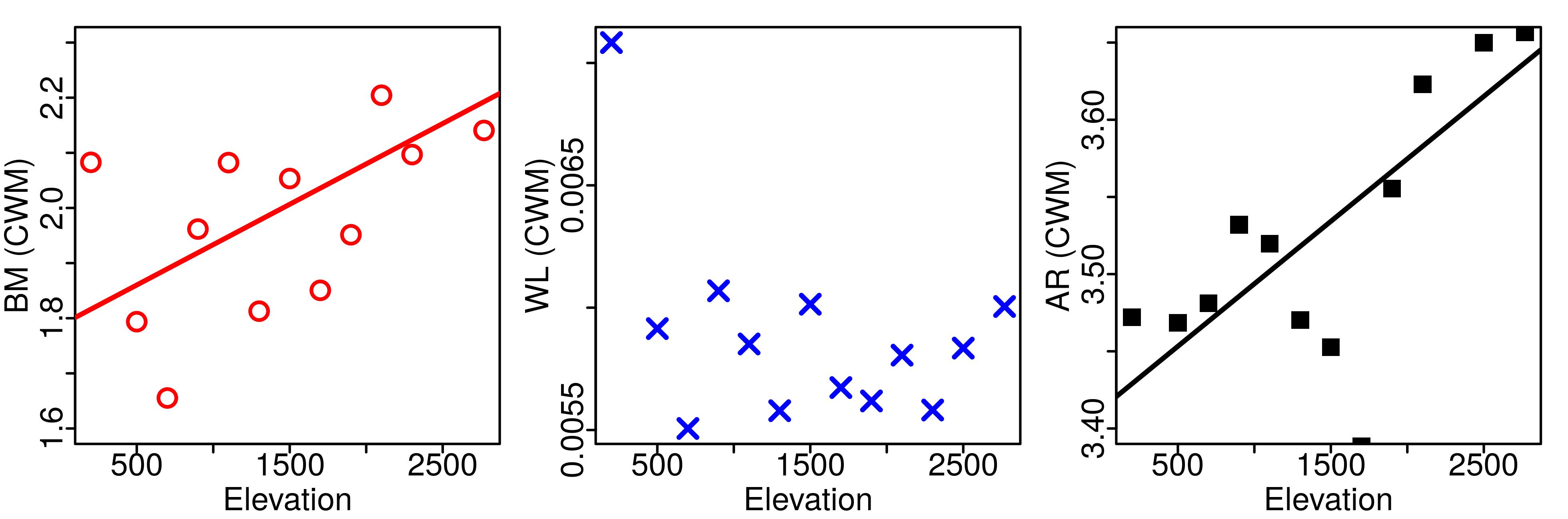
**

**Figure E1: Relationship between hawkmoth community mean trait values and elevation**

The plots show the change in community weighted means of body mass (BM), wing loading (WL) and wing aspect ratio (AR) plotted against elevation. The community mean values were calculated using the population-specific mean trait for each species in a community. The dashed and solid lines indicate regression fits significant at the 90% (p < 0.1) and 95% level (p < 0.05) levels, respectively. The regression parameters are in Table E1.

**
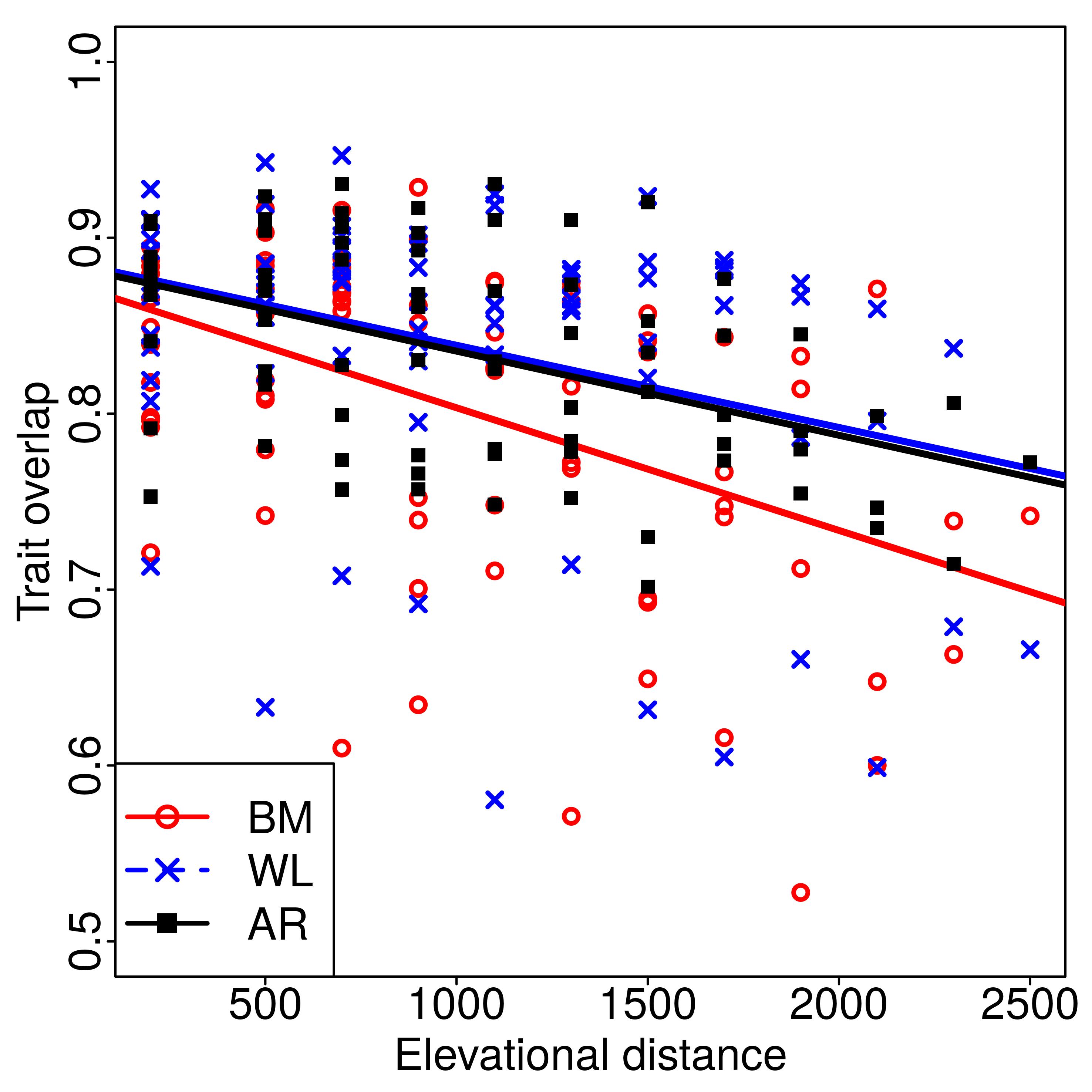
**

**Figure E2. Relationship between hawkmoth community trait overlap and elevational distance**

The plot shows the scatter and the regression lines for the relationship between the overlap in trait distribution functions for pairs of communities and the elevational distance between them. The three traits plotted are body mass (BM), wing loading (WL), and wing aspect ratio (AR). The overlap for a pair of communities was calculated from the area of intersection of their trait kernel density distributions. The solid lines indicate regression fits significant at the 95% level (p < 0.05) levels. The regression parameters are in Table E1.


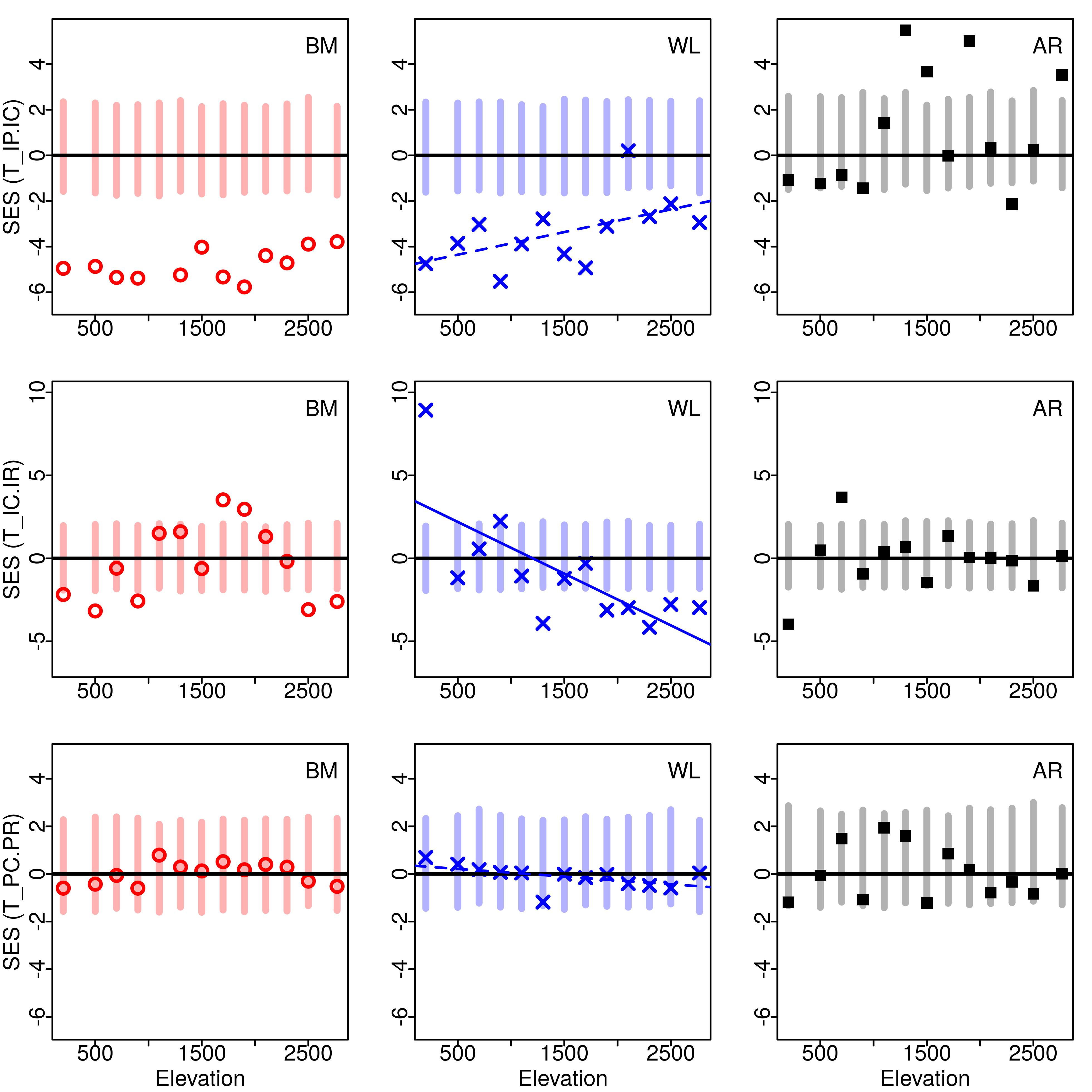
**Figure E3: *T-statistics* of hawkmoth functional traits across an elevation*al* gradient.** The plots show the standardised effect sizes (SES) of *T-statistics* metrics for body mass (BM), wing loading (WL), and wing aspect ratio (AR) for each of the 13 elevational communities. The vertical bars represent the 95% distribution of simulated null communities, and the dots are the observed values. The metrics are variance ratios of (a) TIP/IC: intra-population to intra-community (b) TIC/IR: intra-community to regional, assessed using individual trait values, and (c) TPC/PR: intra-community to regional, assessed using population mean values. The dashed and solid lines indicate regression fits significant at the 90% (p < 0.1) and 95% level (p < 0.05) levels, respectively. The regression parameters are in Table 2.

**References**

1. McGeachie, W.J., 1989. The effects of moonlight illuminance, temperature and wind speed on light-trap catches of moths. *Bulletin of Entomological Research*, *79*(2), pp.185-192.
2. Beck, E., Bendix, J., Kottke, I., Makeschin, F. and Mosandl, R. eds., 2008. *Gradients in a tropical mountain ecosystem of Ecuador* (Vol. 198). Springer Science & Business Media.
3. Schulze, C.H. and Fiedler, K., 2003. Vertical and temporal diversity of a species-rich moth taxon in Borneo. *Arthropods of tropical forests: spatio-temporal dynamics and resource use in the canopy*, pp.69-85.
4. Willott, S.J., 2001. Species accumulation curves and the measure of sampling effort. *Journal of applied ecology*, *38*(2), pp.484-486.
5. Melo, D., Garcia, G., Hubbe, A., Assis, A.P. and Marroig, G., 2015. EvolQG-An R package for evolutionary quantitative genetics. *F1000Research*, *4*.
